# Targeting ESR1 restores SQSTM1-dependent autophagy and sensitizes ER-positive breast cancer to oxidative and radiation stress

**DOI:** 10.1038/s41420-025-02755-8

**Published:** 2025-10-07

**Authors:** Yi-Fang Yang, Zhao-Jing He, Han-Hsi Kuo, Yu-Yu Lin, Cheorl-Ho Kim, Huei-Yu Cai, Chi-Long Chen, Michael Hsiao, Ying-Chung Chen, Peter Mu-Hsin Chang, Yu-Chan Chang

**Affiliations:** 1 Department of Medical Education and Research, Kaohsiung Veterans General Hospital, Kaohsiung, Taiwan; 2 Department of Biomedical Imaging and Radiological Sciences, National Yang Ming Chiao Tung University, Taipei, Taiwan; 3 Molecular and Cellular Glycobiology Unit, Department of Biological Sciences, SungKyunKwan University, Suwon, Gyunggi-Do Republic of Korea; 4 Department of Pathology, College of Medicine, Taipei Medical University and Taipei Medical University Hospital, Taipei, Taiwan; 5 Genomics Research Center, Academia Sinica, Taipei, Taiwan; 6 Department of Physiology and Biophysics, National Defense Medical University, Taipei, Taiwan; 7 Institute of Biopharmaceutical Sciences, National Yang Ming Chiao Tung University, Taipei, Taiwan; 8 Department of Oncology, Taipei Veterans General Hospital, Taipei, Taiwan

**Keywords:** Breast cancer, Cell death, Double-strand DNA breaks

## Abstract

Estrogen receptor-positive (ER⁺) breast cancer is commonly treated with hormone therapy; however, these tumors frequently develop drug resistance and exhibit poor responses to radiotherapy. To investigate the molecular basis of therapy resistance, we explored the role of estrogen receptor alpha (ESR1) in modulating sensitivity to oxidative and radiation stress. Through integrative analysis of publicly available datasets, we identified ESR1 as a key molecular marker associated not only with breast cancer classification but also with radiosensitivity. In ER⁺ breast cancer cell lines, higher endogenous ESR1 expression correlated with increased resistance to ionizing radiation. Functional studies using ESR1 overexpression and knockdown models revealed that depletion of ESR1 sensitized cells to radiation-induced DNA damage, impaired DNA repair efficiency, and reduced clonogenic survival. Notably, we found that the ESR1–SQSTM1 (p62) interaction impairs autophagic flux, contributing to treatment resistance. Mechanistically, ESR1 translocates to the cytoplasm and binds to SQSTM1, thereby disrupting autophagosome maturation. Furthermore, estradiol enhances ESR1 phosphorylation and its affinity for SQSTM1, reinforcing this inhibitory effect on autophagy and promoting resistance to radiation. Our findings uncover a previously unrecognized ESR1–SQSTM1 axis that governs autophagy and redox response in ER⁺ breast cancer. Targeting this pathway may restore sensitivity to radiotherapy and offer a new therapeutic strategy. Assessment of ESR1 expression and autophagy activity may serve as predictive biomarkers for treatment response in ER⁺ breast cancer patients.

## Introduction

Breast cancer is the most commonly diagnosed cancer in women worldwide [1]. In Taiwan, breast cancer is the third leading cause of cancer-related deaths among women, according to the Ministry of Health and Welfare [2]. In addition, breast cancer occurs at a younger age in Taiwan. The median age of breast cancer diagnosis in Taiwan is between 45 and 49 years, and 16.6% of breast cancer patients in Taiwan are younger than 40 years [3]. The widely accepted classification of breast cancer is based on the expression of the following receptors: estrogen receptor (ER), progesterone receptor (PR), and human epidermal growth factor receptor 2 (HER2) [4]. As a result, breast cancer has four major subtypes: estrogen receptor-positive (ER^+^), progesterone receptor-positive (PR^+^), human epidermal growth factor receptor-positive (HER2^+^), and triple-negative breast cancer (TNBC) [5]. In Taiwan, approximately 70% of breast cancer patients have ER^+^ and PR+ subtypes, with HER2^+^ breast cancer accounting for 20-25% and TNBC accounting for 10–15% [6]. The mainstay of locally advanced breast cancer treatment is surgery, with local radiotherapy, systemic hormone therapy, chemotherapy and targeted therapy as adjuvant treatment options. The determination of a treatment plan depends on both the stage and hormone receptor expression levels [7]. Becaure hormone receptors promote the growth and spread of cancer cells [8], so hormone therapy drugs are used to reduce or block hormones to slow or stop cancer cell growth [9]. However, up to 40% of patients develop resistance to hormone therapy during treatment [10], often leading to disease recurrence and progression. Therefore, there is an urgent need for new strategies to combat this resistance.

Estrogen receptors (ER) are hormone receptors that are activated by estrogen and include ERα (ERα), ERβ (ERβ), and GPER (GPR30) [11]. ERα and ERβ belong to the nuclear receptor family, while GPER is a membrane receptor. ESR1 encodes ERα, which is mainly expressed in the female breast, ovaries and uterus [12]. The expression of ERα in breast cancer cells (50-80%) is higher than in normal breast cells (10%), and it promotes tumor growth by interacting with estrogen [13]. Approximately 60–80% of breast cancers are ER-positive. As a result, selective ER modulators (SERMs) and selective ER degraders (SERDs) have been developed for hormonal therapy to block ER signaling pathways [14]. SERMs, such as tamoxifen, act as antagonists and inhibit ER-dependent transcription in the mammary gland [15]. SERDs, such as fulvestrant, degrade the ER to prevent its translocation to the nucleus [16]. However, hormone resistance hinders the effectiveness of treatments for ER-positive breast cancer by blocking the effects of estrogen. Research suggests that hormonal resistance occurs due to loss of ER expression and mutations in estrogen receptor 1 (ESR1), typically Y537 and D538 [17, 18].

Radiotherapy has been the standard treatment for locally advanced breast cancer for decades. Radiation therapy over the breast tissue, chest wall, or regional lymph nodes has been approved to reduce the recurrence and prolong survival by several large randomized trials [19–22]. There is no doubt about the importance of radiotherapy in the treatment of breast cancer. The effectiveness of this therapeutic approach is highly dependent on the radiosensitivity of the tumor cells. ESR1 mutations have been shown to contribute to tumor cell resistance to hormonal and radiotherapy, as demonstrated in a recent study [23]. In addition, ER-positive cell lines containing ESR1 mutations have less unrepaired DNA damage after radiotherapy and are therefore resistant to radiotherapy. This suggests that inhibition of ESR1 may have a radiosensitizing effect. However, little research has been conducted to explore the mechanism of ESR1 induced radiation resistance. In this study, we showed that ESR1 may interrupt autophagy via SQSTM1 to protect breast cancer cell from radiation related DNA damage and cause resistance.

## Results

### ESR1 was associated with radioresistant in ER^+^ breast cancer cells

**Fig. 1:**
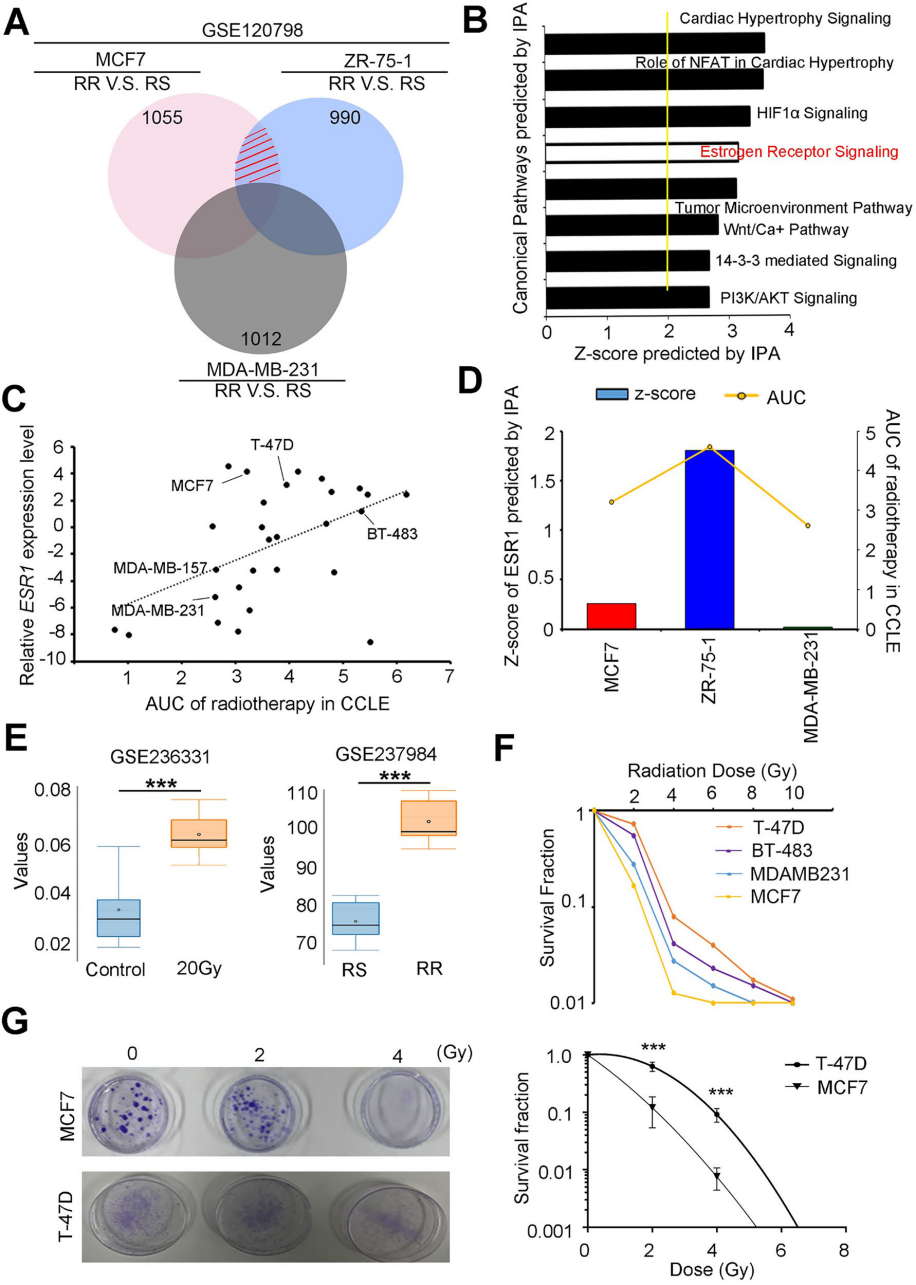
Activation of ESR1 in response to radiation exposure or radioresistance in breast cancer cells. **A** Venn diagram showing common signatures and overlapping regions of the three cellular profiles of GSE120798. **B** Canonical pathways predicted by IPA and sorted by Z-score. **C** The correlation plot between ESR1 expression levels and radioresistance AUC in breast cancer cell lines from CCLE. **D** Z-score of ESR1 activation and its corresponding radioresistance AUC in three breast cancer cell lines (MCF7, ZR-75-1 and MDA-MB-231). **E** Expression level of ESR1 in GSE236331 and GSE237984 datasets. **F** Survival fraction of 4 breast cancer cell lines (T-47D, BT-483, MDA-MB-231 and MCF7) exposed to irradiation. **G** Colony forming ability of MCF7 and T-47D cells with or without dose-dependent irradiation. The data from three independent experiments are presented as the means ± SEM.

To elucidate the mechanisms underlying radioresistance in breast cancer cells, we analyzed multiple publicly available gene expression datasets. Differentially expressed genes (DEGs) were identified by comparing estrogen receptor-positive (ER^+^) cell lines MCF-7 and ZR-75-1, while excluding the ER-negative MDA-MB-231 cell line from dataset GSE120798. Following normalization, DEGs were selected using a fold-change threshold of ≥1.5. For illustration, dataset GSE120798 was used to classify cells into radioresistant (RR) and radiosensitive (RS) groups, leading to the identification of DEGs specific to the RR phenotype. Common gene signatures in RR cells were further refined by excluding changes found in RS groups (Fig. 1A). Ingenuity Pathway Analysis (IPA) of these DEGs revealed significant activation of pathways associated with estrogen receptor 1 (ESR1), hypoxia-inducible factor 1-alpha (HIF-1α), and the tumor microenvironment (Fig. 1B). To validate these findings, we examined ESR1 expression across breast cancer cell lines from the Cancer Cell Line Encyclopedia (CCLE) and correlated this with their respective radiation responses. A significant positive correlation was observed between ESR1 expression and radioresistance (Fig. 1C). Notably, ESR1 activation was detected in radioresistant MCF7 and ZR-75-1 cells within two hours of 20 Gy irradiation, while no such activation was observed in MDA-MB-231 cells. In silico analysis further supported these observations, as ESR1 consistently exhibited a Z-score >1 (indicative of activation) and a high area under the curve (AUC) for radioresistance across various ER+ breast cancer cell lines (Fig. 1D). Additional datasets confirmed that ESR1 expression is upregulated either following radiation exposure or in models of acquired radioresistance (Fig. 1E). To further validate these findings, we analyzed a panel of ER+ breast cancer cell lines—including MCF7, ZR-75-1, BT-483, and T-47D—to assess their survival fraction post-irradiation and corresponding endogenous ESR1 protein levels (Fig. 1F). Two representative cell lines (T-47D and MCF7) were selected from the panel and exhibited different radiation responses and DNA damage statuses (Fig. 1G). Collectively, our data suggest that ESR1 expression is associated with, and may serve as a predictive marker of, radiation sensitivity in ER+ breast cancer cell lines.

### ESR1 protected DNA damage and activated double-strand break (DSB) repair mechanisms after irradiation

**Fig. 2:**
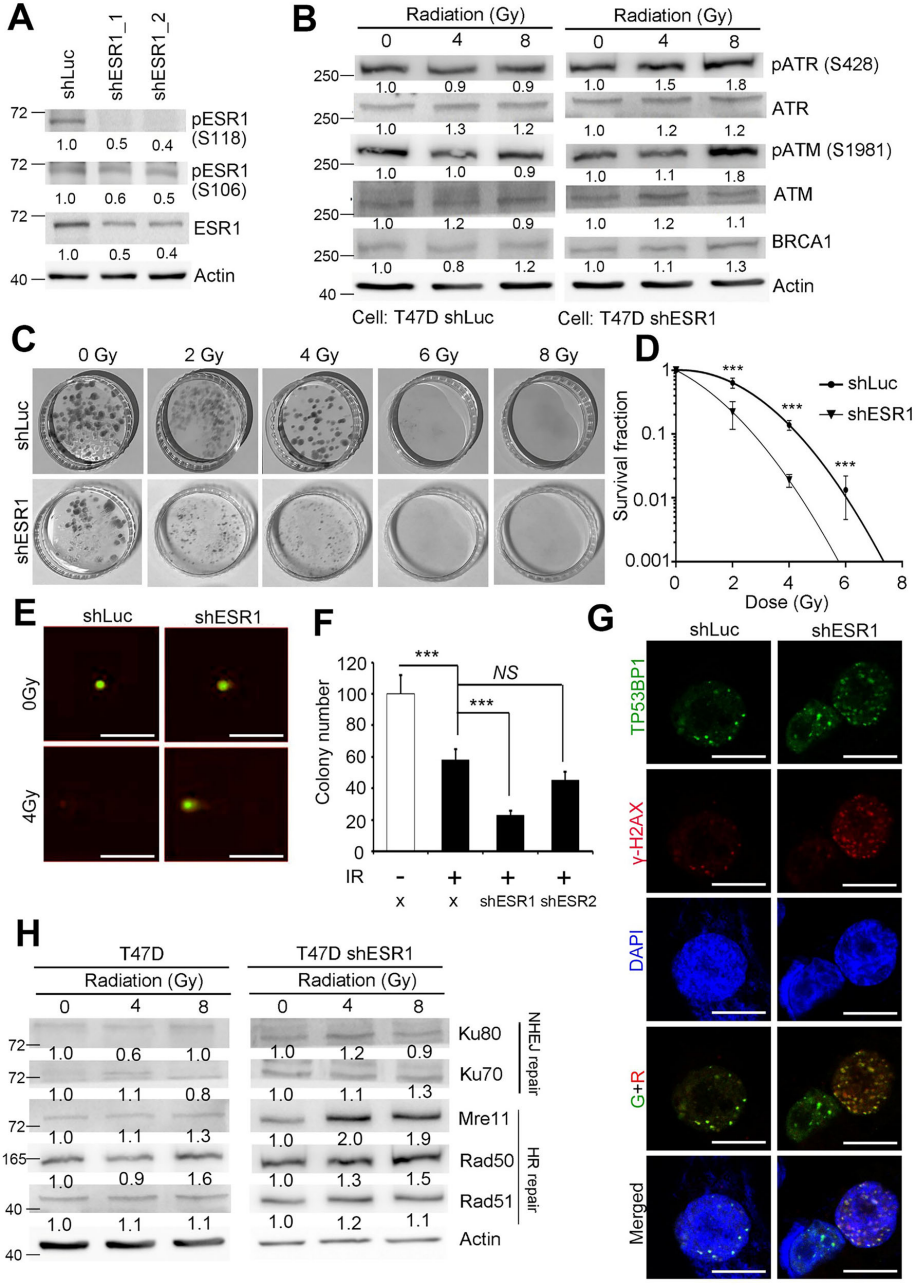
Loss of ESR1 increased DNA damage and DNA repair after irradiation. **A** Protein levels of ERα and p-ERα (S118 and S106) in T47D with and without ESR1 knockdown. Actin was used as internal control. **B** Protein levels of ATR, p-ATR (S428), ATM, p-ATM (S1981) and BRCA1 in T47D with or without ESR1 knockdown in a radiation dose-dependent manner. Actin was used as internal control. **C** Evaluation of DNA damage level of ESR1 knockdown model by COMET assay. **D** Colony forming ability of T47D ESR1 knockdown model. **E** Survival fraction of ESR1 knockdown model exposed to irradiation. **F** Quantification of colony number of T47D with or without combined shESR1/shESR2 exposed to irradiation. **G** Images of TP53BP1 and γ-H2AX in T47D ESR1 knockdown model by immunofluorescence. DAPI was used as internal control. **H** Protein levels of Ku80, Ku70, Mre11, Rad50 and Rad51 in T47D with or without ESR1 knockdown in a radiation dose-dependent manner. Actin was used as internal control. The data from three independent experiments are presented as the means ± SEM.

Given that we observed an upregulation of ESR1 expression following irradiation exposure, we established an ESR1 knockdown model in estrogen receptor-positive (ER^+^) breast cancer cells. In these knockdown models, both the total protein level of ESR1 and its phosphorylation status were significantly reduced (Fig. 2A). Additionally, several DNA damage indicators were observed in the ESR1 knockdown model upon receiving radiation (Fig. 2B). Colony formation assays demonstrated that ESR1 knockdown in ER^+^ breast cancer cells increased sensitivity to ionizing radiation (IR) exposure (Fig. 2C, D). To further investigate the impact on DNA damage response, we conducted a COMET assay, which revealed a significantly greater DNA damage response in ESR1 knockdown cells compared to controls (Fig. 2E). Moreover, we investigated the potential role of estrogen receptor 2 (ESR2), given that several studies have suggested its coordination with ESR1. However, our results showed that ESR2 did not significantly affect the radiation response in the models exposed to IR (Fig. 2F). To assess the impact on DNA damage repair, we evaluated the formation of foci associated with TP53BP1 and γ-H2AX. In ESR1 knockdown cells, the number of these foci was significantly increased compared to the control group, indicating enhanced DNA damage (Fig. 2G). We also examined the dose-dependent response to irradiation and found that the DNA damage response-related genes ATM/ATR were activated, with phosphorylation of these proteins being upregulated. Furthermore, downstream genes such as BRCA1 were also activated under irradiation (Fig. 2B). In parallel, we observed significant changes in the expression of non-homologous end joining (NHEJ)-related genes, Ku70 and Ku80, which are involved in DNA double-strand break repair. Conversely, genes related to homologous recombination (HR), including Rad50, Mre11, and Rad51, displayed consistent responses in our model (Fig. 2H). These findings suggest that ESR1 plays a role in conferring resistance to DNA damage induced by irradiation.

**Fig. 3:**
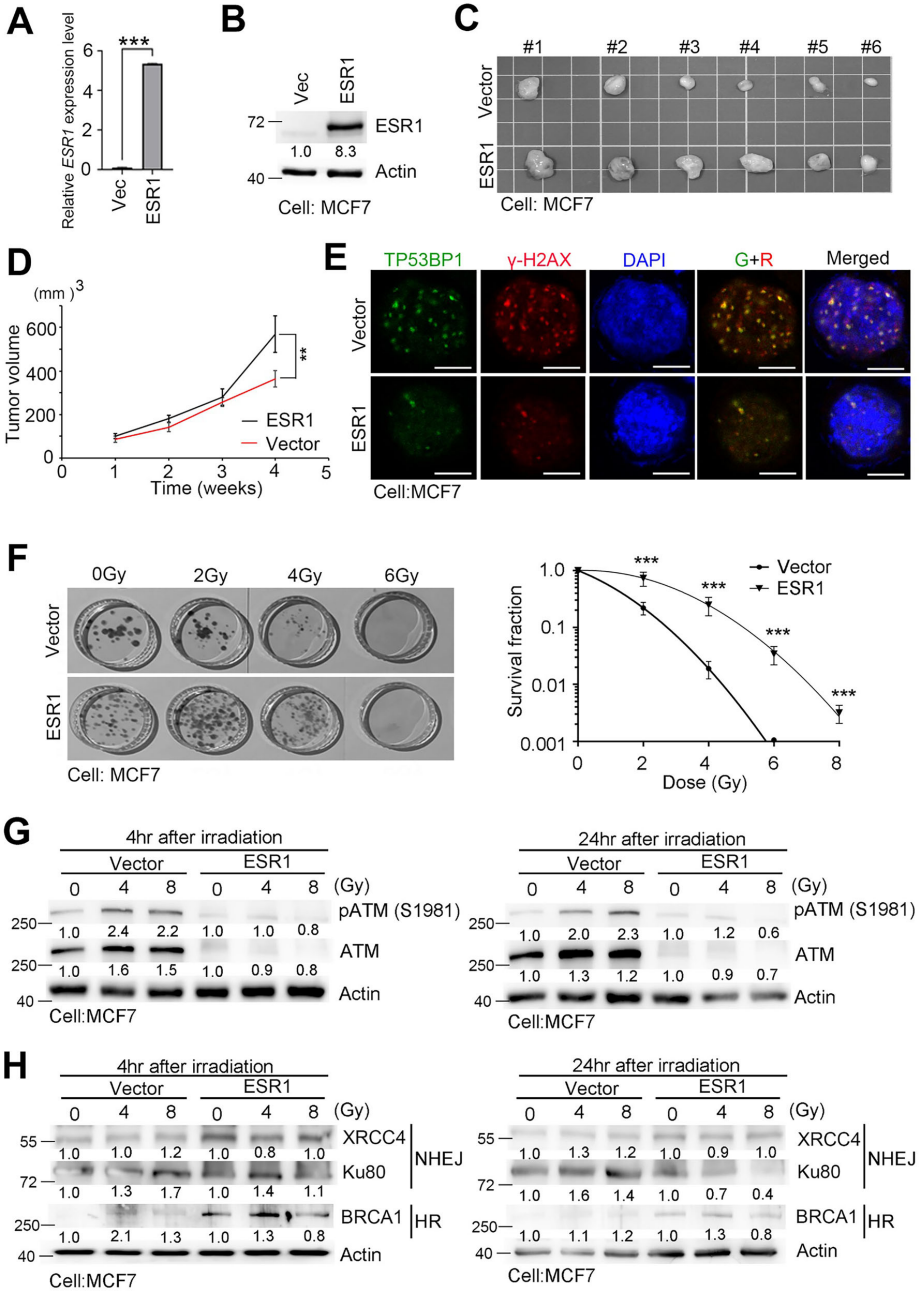
ESR1 promoted tumorigenesis and prevents radiation-induced DNA damage. **A** Endogenous ESR1 expression in MCF-7 cells. **B** ESR1 expression level in MCF-7 with and without ESR1 overexpression. **C** Overview of the xenograft tumor of ESR1 models. **D** Growth curve of tumorigenicity in ESR1 overexpression models. **E** Images of TP53BP1 and γ-H2AX in MCF7 ESR1 overexpression model by immunofluorescence. DAPI was used as an internal control. **F** Representation of colony formation and survival fraction of ESR1 overexpression model exposed to irradiation. **G** Protein levels of ATM and p-ATM (S1981) in MCF-7 with or without ESR1 overexpression in a radiation dose-dependent manner. Actin was used as an internal control. **H** Protein levels of XRCC4, Ku80 and BRCA1 in MCF-7 with or without ESR1 overexpression in a radiation dose-dependent manner. The data from three independent experiments are presented as the means ± SEM.

We also investigated the effect of ESR1 in an overexpression model. To this end, we introduced ESR1 overexpression into a radiosensitive ER+ breast cancer cell line, MCF7, which has low endogenous ESR1 expression (Supplementary Fig. 1). In addition to confirming the successful expression of ESR1, we also validated the phenotypic changes in an animal model. Both in vitro expression levels and in vivo tumor growth were significantly increased in the ESR1 overexpression model (Fig. 3A–D). In contrast to the ESR1 knockdown model, our data suggest that ESR1 elevates the threshold for DNA damage and impedes subsequent DNA repair mechanisms. To explore this further, we performed a foci formation assay, where both TP53BP1 and γ-H2AX foci were suppressed in the ESR1 overexpression model (Fig. 3E). Furthermore, ESR1 overexpression conferred acquired resistance to ionizing radiation (IR) exposure (Fig. 3F). Focusing on molecules related to DNA double-strand break (DSB) repair and associated repair mechanisms, we observed that the expression of key DNA repair proteins, including ATM, XRCC4, and Ku80 (involved in non-homologous end joining, NHEJ), as well as BRCA1 (involved in homologous recombination, HR), was compromised in the ESR1 overexpression model (Fig. 3G, H). These findings are consistent with those observed in the ESR1 knockdown model.

TP53 is considered a key contributor to both DNA damage and repair. Since the two cell models had different genetic backgrounds regarding TP53 expression (T-47D: L194F, MCF7: wild-type), we created a knockdown model in MCF7 cells to evaluate the radiation response. After confirming the efficiency of TP53 knockdown and the expression of downstream factors, we found that TP53 depletion only slightly increased the radiation response (Supplementary Fig. 2). This suggests that TP53 may not be a major contributor in our model. To mitigate cellular heterogeneity, we also established a syngeneic model in T47D cells that included both existing knockdown sublines and novel overexpression sublines (Supplementary Fig. 3). We determined that ESR1 overexpression promoted phosphorylation and enhanced radioresistance compared to other models.

### Autophagy was regulated by ESR1 in response to radiation-induced DNA damage

**Fig. 4:**
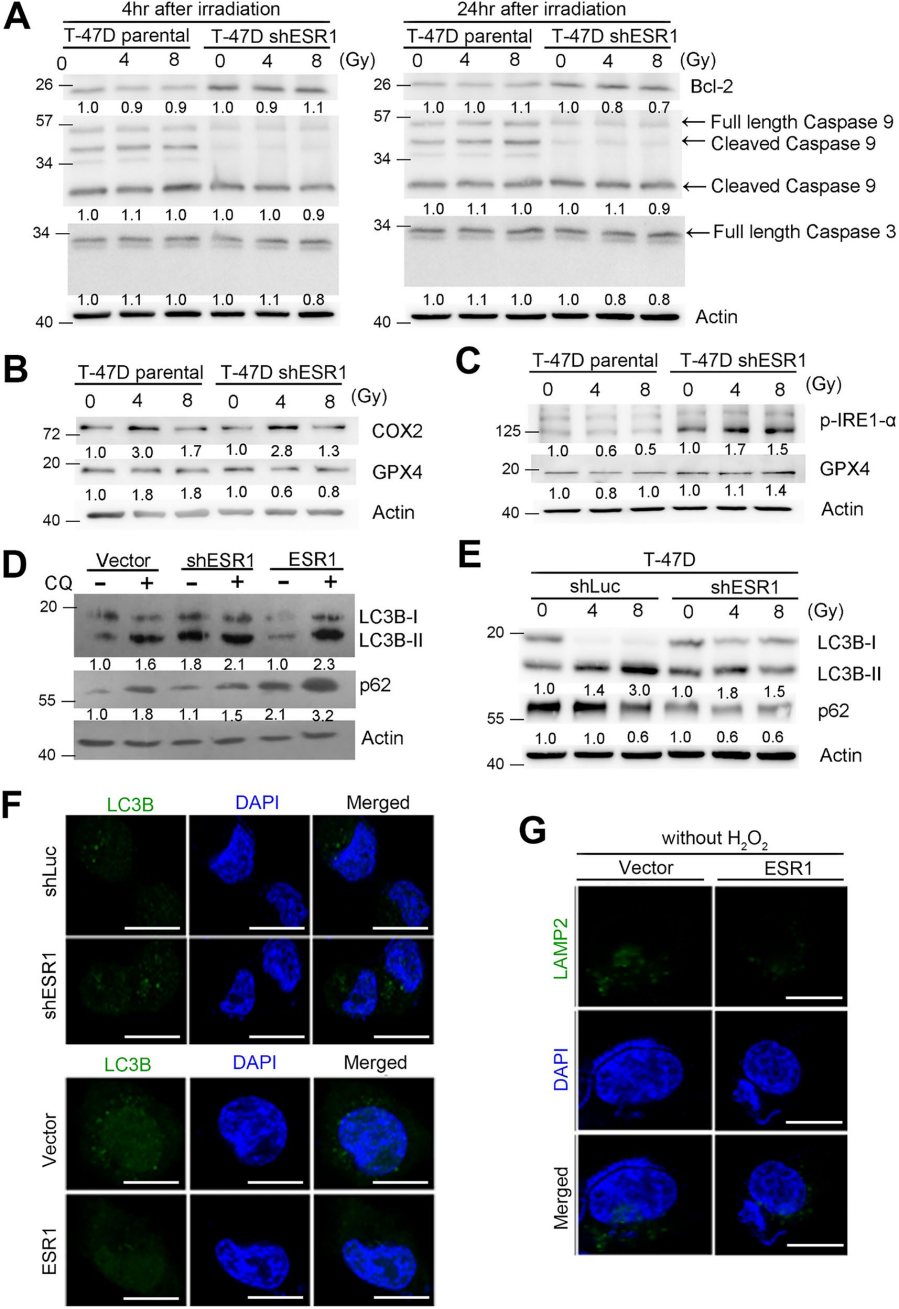
ESR1 resistance to radiation increased ER stress and autophagy formation. **A** Protein levels of Bcl-2, caspase 9 (full length/cleaved) and caspase 3 in T47D with or without ESR1 knockdown in a radiation dose-dependent manner. Actin was used as internal control. **B** Protein levels of COX2 and GPX4 in T47D with or without ESR1 knockdown in a radiation dose-dependent manner. Actin was used as internal control. **C** Protein levels of p-IRE1-α and IRE1-α in T47D with or without ESR1 knockdown in a radiation dose-dependent manner. Actin was used as internal control. **D** Protein levels of LC3B (I/II) and p62 in T47D isogenic model with or without CQ treatment. Actin was used as internal control. **E** Protein levels of LC3B (I/II) and p62 in T47D with or without ESR1 knockdown in a radiation dose-dependent manner. Actin was used as internal control. **F** Images of LC3B in ESR1 overexpression or knockdown model by immunofluorescence, respectively. DAPI was used as internal control. **G** Images of LAMP2 in ESR1 overexpression model by immunofluorescence. DAPI was used as internal control. The data from three independent experiments are presented as the means ± SEM.

When radiation induces severe and irreparable DNA damage, cell death programs such as apoptosis, autophagy, and necrosis are activated [24–26]. To confirm these events, we analyzed several key molecules in the cellular model. We assessed the expression levels of Bcl-2 and the full-length/cleaved forms of caspase-3 and caspase-9. Although baseline expression levels of these molecules varied between the control and ESR1 knockdown models, they did not change significantly following irradiation. Both low and high doses of irradiation, as well as short-term and long-term exposures, exhibited similar trends (Fig. 4A and Supplementary Fig. 4). Additionally, we examined markers of ferroptosis, such as COX2 and GPX4, but found no significant changes in our cell model after irradiation (Fig. 4B). We hypothesize that irradiation may trigger endoplasmic reticulum (ER) stress due to the generation of reactive oxygen species (ROS), primarily through mitochondrial membrane potential disruption, leading to protein misfolding and unfolding. Indeed, we observed increased phosphorylation of IRE1-α in the ESR1 knockdown group following irradiation, compared to the control group (Fig. 4C). To further investigate the involvement of autophagy, we examined autophagy-related markers, as autophagy is activated to clear misfolded proteins. In the T-47D isogenic model, treatment with chloroquine (CQ) induced accumulation of LC3B and increased p62, thereby blocking autophagic flux. ESR1 overexpression significantly increased LC3B and p62, thereby also blocking autophagic flux (Fig. 4D). In the ESR1 knockdown model, we found that radiation did not block autophagic flux (Fig. 4E). In contrast, the ESR1 overexpression model showed opposite trends for these markers (Supplementary Fig. 5). To track the localization of LC3B and LAMP2, our results confirmed that ESR1 overexpression inhibits autophagosome formation (Fig. 4F, G and supplementary Fig. 6). Taken together, these data suggest that ESR1 attenuates ER stress and autophagy-dependent cell death in ER^+^ breast cancer cells following irradiation.

### ESR1 contributed its function to combat indirect ionizing radiation

Ionizing radiation can directly interact with fundamental cellular targets, such as DNA, by transferring energy that causes the ionization of atoms. This process induces biological alterations through direct action. In addition, reactive oxygen species (ROS), including hydrogen atoms (H^+^), hydroxyl radicals (^•^OH), superoxide anions (O_2_^•−^), and hydrogen peroxide (H_2_O_2_), can interact with DNA and induce biological modifications through indirect effects [27]. To investigate whether H_2_O_2_ induces similar effects to ionizing radiation, we included hydrogen peroxide in our experimental model.

**Fig. 5:**
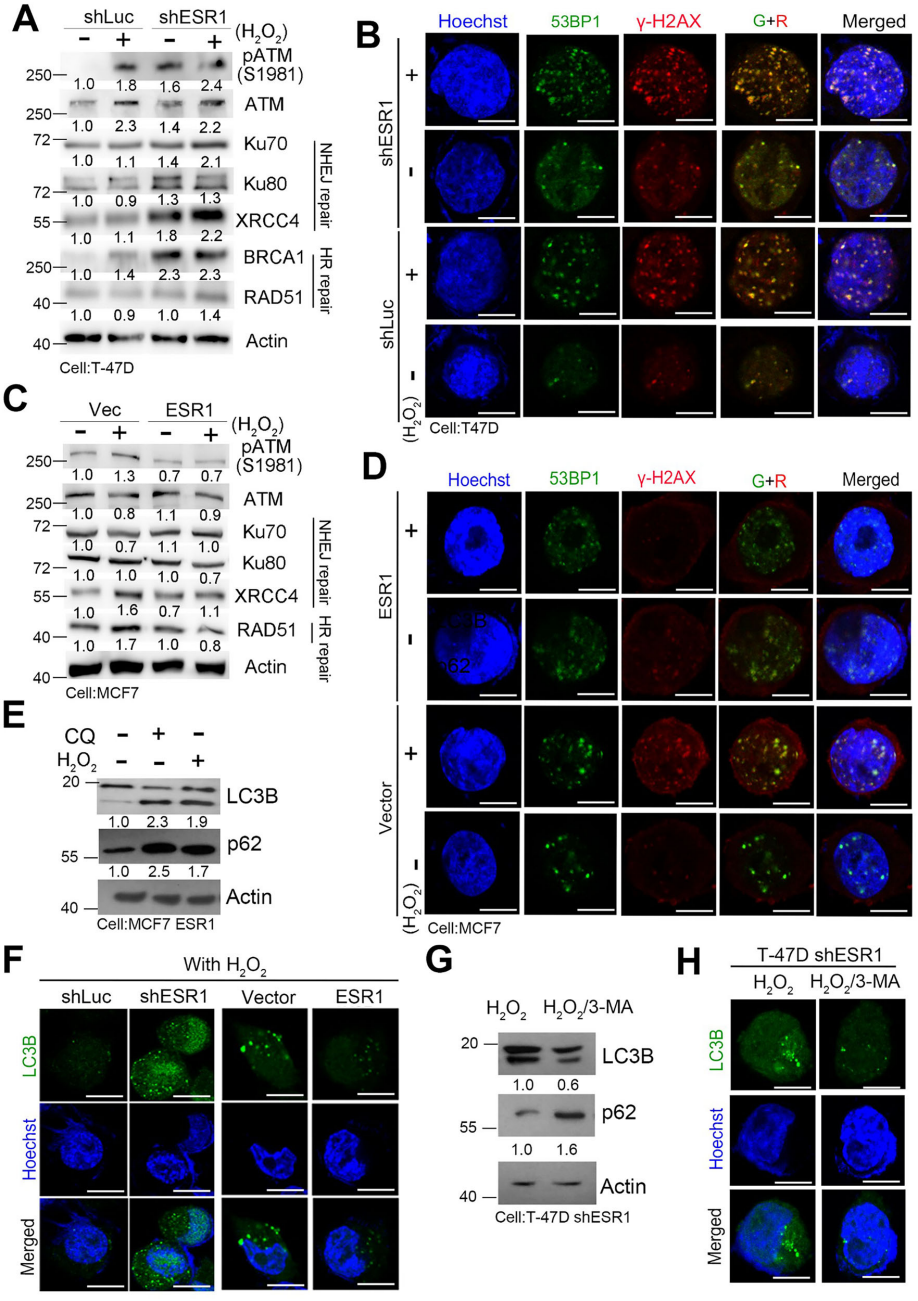
ESR1 was responsible for DNA damage caused by irradiation and ROS overproduction. **A** Protein levels of ATM, p-ATM (S1981), Ku70, Ku80, XRCC4, Rad51 and BRCA1 in T47D with or without ESR1 knockdown by H2O2 treatment. Actin was used as an internal control. **B** Images of TP53BP1 and γ-H2AX in T47D ESR1 knockdown model by H_2_O_2_ treatment by immunofluorescence. Hoechst was used as an internal control. **C** Protein levels of ATM, p-ATM (S1981), Ku70, Ku80, XRCC4 and Rad51 in MCF7 with or without ESR1 overexpression by H_2_O_2_ treatment. Actin was used as an internal control. **D** Images of TP53BP1 and γ-H2AX in MCF7 ESR1 overexpression model by H_2_O_2_ treatment via immunofluorescence. Hoechst was used as an internal control. **E** Protein levels of LC3B (I/II) and p62 in T47D ESR1 knockdown model with or without CQ/ H_2_O_2_. Actin was used as an internal control. **F** Images of LC3B in ESR1 overexpression and knockdown model by H_2_O_2_ treatment via immunofluorescence. Hoechst was used as an internal control. **G** Protein levels of LC3B (I/II) and p62 in T47D ESR1 knockdown model treated with H_2_O_2_ with or without 3-MA. Actin was used as an internal control. **H** Images of LC3B in the MCF7 ESR1 overexpression model were taken after treatment with H₂O₂ for one hour and with or without 3-MA for three hours. Hoechst was used as the internal control. The data from three independent experiments are presented as the means ± SEM.

Our results indicate that H_2_O_2_ treatment leads to an increase in the expression of phosphorylated ATM. Following DNA double-strand breaks, repair machinery associated with non-homologous end joining (NHEJ) (including Ku70, Ku80, and XRCC4) and homologous recombination (HR) (including BRCA1 and Rad51) is activated (Fig. 5A). Given that DNA damage is known to elevate the levels of γ-H2AX and phosphorylated tumor protein p53 binding protein 1 (TP53BP1), we performed immunofluorescence analysis to examine the formation of TP53BP1 and γ-H2AX foci. Our results demonstrate that exposure to H_2_O_2_ significantly enhances the formation of these foci (Fig. 5B). Importantly, we observed that the ESR1 knockdown group treated with H_2_O_2_ exhibited the highest number of lesions, providing evidence that ESR1 knockdown exacerbates H_2_O_2_-induced DNA damage. In contrast, ESR1 overexpression models showed complementary results, where ESR1 appeared to prevent DNA damage, repair, and lesion formation upon H_2_O_2_ treatment (Fig. 5C, D). Regarding autophagy, our data revealed that p62 expression increased in ESR1-overexpressing cells and that autophagic flux was inhibited regardless of treatment with the inhibitors CQ or H_2_O_2_ (Fig. 5E). Similarly, LC3B fluorescence signaling and localization exhibited consistent patterns with H_2_O_2_ and irradiation exposure (Fig. 5F). Adding the autophagy inhibitor 3-methyladenine (3-MA) resulted in enhanced p62 expression, blocked autophagic flux, and reduced LC3B puncta levels in ESR1 knockdown cells (Fig. 5G, H). These findings suggest that ESR1 may directly or indirectly modulate DNA damage induced by ionizing radiation and hydrogen peroxide treatment.

### ESR1 attenuated p62 phosphorylation status and suppressed autophagy events

**Fig. 6:**
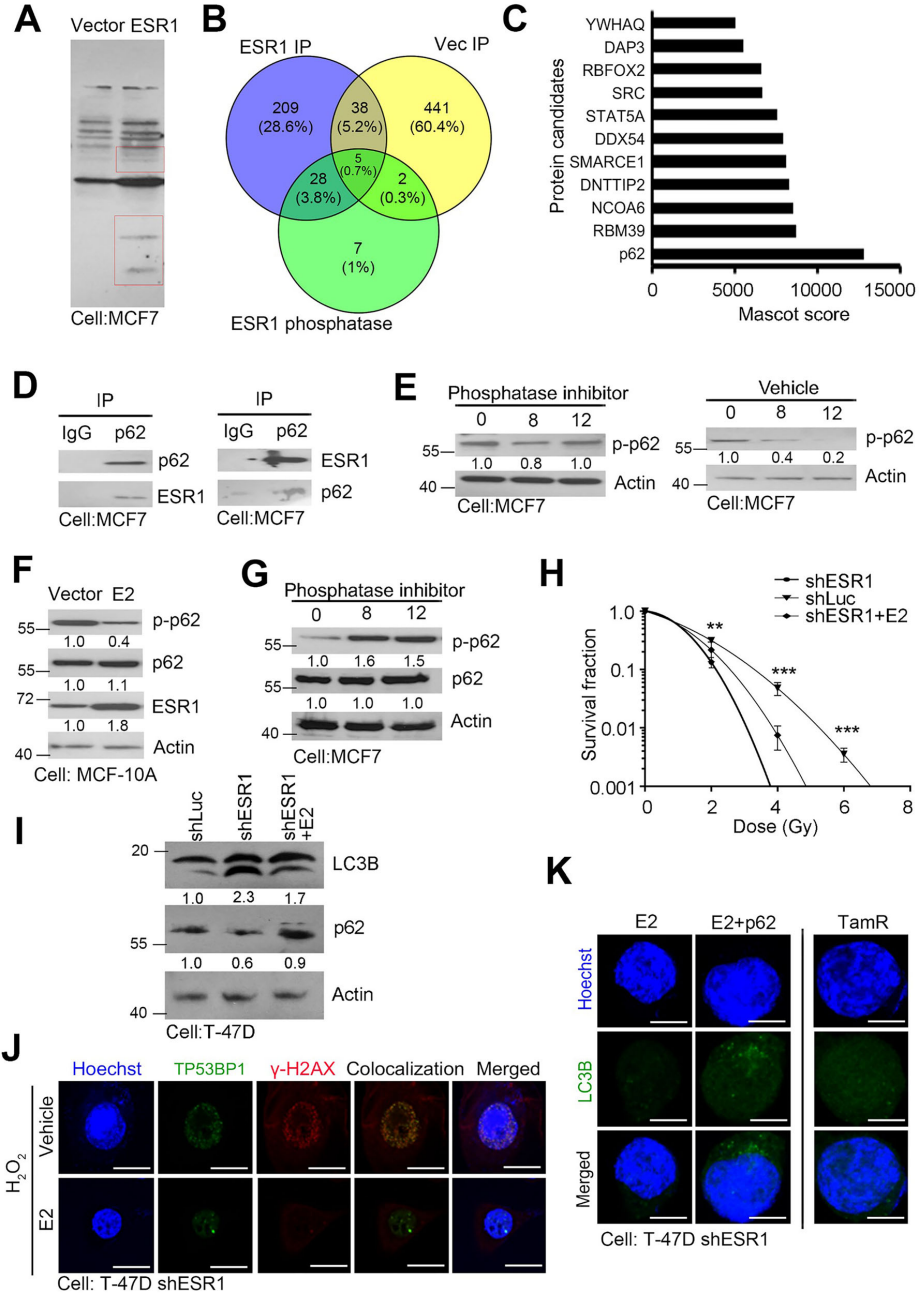
ESR1 interfered with post-translational modification of p62 and autophagosome formation. **A** Electrophoretic separation and Coomassie blue staining of MCF7 with and without ESR1 overexpression. **B** Venn diagram showing the overlapping molecules between ESR1 IP, vector IP and ESR1 with MG-132 treatment. **C** Top ranking of selected molecules from ESR1-based proteomics profiles. **D** Interaction affinity between p62 and ESR1 by pull-down assay in ESR1 overexpression model. **E** Protein levels of p62 in MCF7 ESR1 overexpression by MG-132 treatment. Actin was used as internal control. **F** Protein levels of phosphor-p62, p62 and ESR1 in MCF7 cells with and without E2 treatment. Actin was used as internal control. **G** Interaction affinity between p62 and ESR1 by pull-down assay after E2 treatment. **H** Survival fraction of ESR1 knockdown model with or without E2 treatment followed by irradiation. (**I**) Protein levels of LC3B and p62 in T-47D ESR1 isogenic model. Actin was used as internal control. (**J**) Images of TP53BP1 and γ-H2AX in T47D ESR1 knockdown model by E2 treatment combined with H2O2 by immunofluorescence. Hoechst was used as internal control. **K** Images of LC3B in T47D ESR1 knockdown model by E2 treatment, E2 combined with p62 overexpression and TamR via immunofluorescence. Hoechst was used as internal control. The data from three independent experiments are presented as the means ± SEM.

To explore the potential mechanisms by which ESR1 influences DNA damage and autophagy, we hypothesized that ESR1 may modulate molecules abundant in ER^+^ breast cancer cells. We examined protein expression levels and observed differences in ESR1 overexpression compared to controls (Fig. 6A). To further investigate, we performed mass spectrometry to identify potential interacting components. We selected the top-ranked molecules that could directly bind to ESR1 and enhance the stability of the interaction in ESR1 immunoprecipitation (IP), vector IP, and ESR1 combined with MG132 (Fig. 6B). Among these molecules, we identified p62, a key factor in autophagy, known to undergo ubiquitination and phosphorylation before being incorporated into autophagosomes [28] (Fig. 6C). Our results demonstrate that ESR1 and p62 form a direct protein-protein interaction in breast cancer cells (Fig. 6D). Moreover, this interaction appears to influence the phosphorylation status of p62. We observed higher levels of p62 phosphorylation when a phosphatase inhibitor was added (Fig. 6E). We propose that ESR1 binding to p62 may interfere with the post-translational modifications of p62, thereby reducing autophagic processing.

**Fig. 7:**
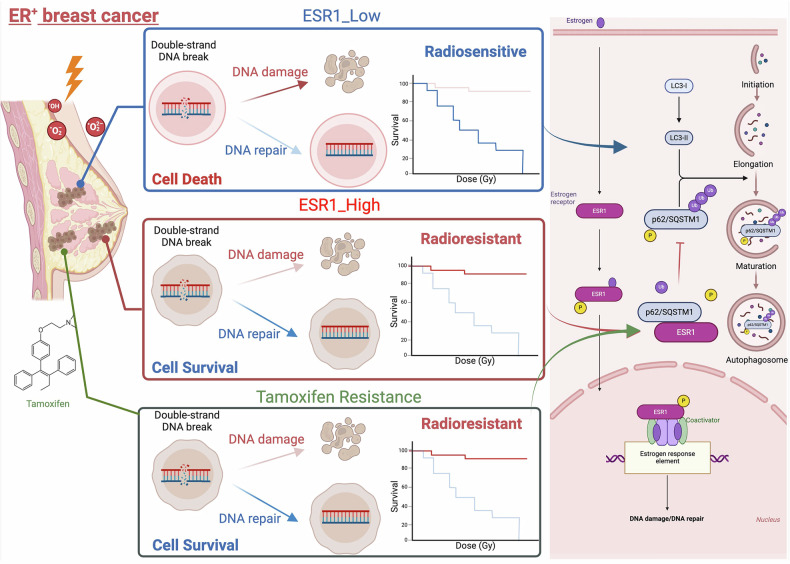
Schematic model of ESR1 resistance to radiation or oxidative DNA damage in ER^+^ breast cancer.

To further investigate the above events, we simulated the ER^+^ breast cancer cell model by treating cells with estradiol (E2). We observed that E2 treatment increased ESR1 expression while decreasing the phosphorylation of p62 (Fig. 6F). Similarly, the phosphorylation of p62 could be restored by the addition of phosphatase inhibitors (Fig. 6G). In terms of ESR1 expression, knockdown of ESR1 increased radiosensitivity, whereas E2 treatment re-inhibited autophagic flux and restored radioresistance in the same model (Fig. 6H, I and Supplementary Fig. 7). Under identical conditions, the E2-treated group also exhibited increased resistance to DNA damage, with a reduction in the co-localization foci of TP53BP1/γ-H2AX (Fig. 6J). Additionally, the LC3B signal was lower in the E2 group, and when p62 overexpression re-sensitized the cells, autophagic activity was restored (Fig. 6K). In clinical practice, some patients develop resistance to tamoxifen upon the use of ESR1 inhibitors [29]. In the tamoxifen-resistant cell line we included, we observed a significant impairment of autophagy (Fig. 6J). Clinically, both ESR1 and SQSTM1 are overexpressed in luminal A/B subtypes, and they form a positive correlation in ER+ breast cancer patients (Supplementary Fig. 8, 9). Taken together, these findings suggest that ESR1 is a crucial factor that interferes with p62 phosphorylation, influencing both autophagy and double-strand break (DSB) repair following radiation (Fig. 7).

## Discussion

Several forms of cell death are observed during radiation exposure, including apoptosis, ferroptosis, autophagy, and cell cycle arrest [30]. When radiation induces an excessive number of DNA double-strand breaks (DSBs) that cannot be repaired, cells will bypass homologous recombination (HR) and non-homologous end joining (NHEJ) repair pathways, ultimately leading to cell death. In this study, we focused on apoptosis and found that key apoptotic markers were not activated, as evidenced by the absence of cleaved caspases. Recently, ferroptosis has been reported to be upregulated in response to reactive oxygen species (ROS) and radiation [31]. It has been shown that downregulation of ESR1 and SLC7A11 can enhance irradiation-induced ferroptosis [32]. However, in our study, we did not observe significant changes in the expression of SLC7A11 and GPX4. Ferroptosis is known to involve three main pathways: the Fenton reaction, lipid peroxidation, and the cystine/glutamate transporter [33]. Therefore, it is crucial to assess lipid peroxidation and transferrin receptor levels to draw more definitive conclusions. Future research will further investigate these mechanisms in detail.

Autophagy is a complex process that can be divided into five steps: phagophore formation, phagophore elongation, autophagosome completion, lysosome fusion, and degradation. During phagophore formation, both the phosphorylation of p62 and damaged mitochondria contribute to the formation of p62 bodies, which are then recruited to the phagophore. Therefore, under conditions of high oxidative stress or ROS induced by radiation or H_2_O_2_, the phosphorylation state and subcellular localization of p62 are critical for assessing autophagic activity. During phagophore elongation, LC3B-I conjugates with phosphatidylethanolamine (PE) to form LC3B-II. Our findings suggest that ESR1 interacts with p62 to prevent its phosphorylation, thus interfering with both phagophore formation and elongation. Consistent with previous studies, ESR1 has been shown to directly bind to p62 [34, 35]. Interestingly, we also discovered that ESR1 affects the phosphorylation of p62. Although the exact binding sites between these two molecules remain unclear, this interaction is important for further investigation and could potentially be engineered to modulate the autophagic process. Additionally, some studies have suggested that the E3 ligase phosphorylates p62, thereby increasing ESR1 expression by promoting the release of ESR1 from the KEAP1-containing complex [36]. This could serve as a feedback mechanism, whereby ESR1 represses p62 phosphorylation.

ESR1 is recognized as a key molecular marker in breast cancer classification. Previous studies have suggested that ESR1 in triple-negative breast cancer (TNBC) cell lines may also enhance radiosensitivity in cancer cells [37]. In TNBC cell lines, such as MDA-MB-231, ESR1 expression has been shown to increase double-strand breaks and delay repair compared to parental cells. Furthermore, ESR1 expression leads to G2/M phase arrest and increased apoptosis following irradiation. Additionally, ERα has been found to reduce the expression of autophagy-related proteins, indicating that autophagic activity decreases in ER-positive MDA-MB-231 cells after irradiation [32]. In our study, we excluded artificially introduced exogenous ESR1 and instead focused on ER-positive breast cancer cells to model hormone-sensitive breast cancer. Previous research has noted that the relationship between ESR1 and radiotherapy may vary depending on the molecular classification of breast cancer [38]. Importantly, we propose a potential mechanism of radiation resistance in ER-positive breast cancer. Our approach could be applied to clinical samples to identify ESR1 and autophagy-related molecules as predictive biomarkers. Additionally, combining ESR1 and/or autophagy antagonists with radiotherapy may offer future therapeutic strategies.

## Material and Methods

### Cell culture

MCF7, BT-483, ZR-75-1, T-47D, and HEK293T cells were obtained from Academia Sinica (Taipei, Taiwan). MCF7 cells was cultured in MEM medium (Gibco, USA). BT-483 and T-47D cells were cultured in RPMI-1640 medium (Gibco, USA) supplemented with 10% fetal bovine serum (FBS) (Hyclone, USA) and 10% penicillin-streptomycin-glutamine (PSG) (Gibco). HEK293T cells were cultured in Dulbecco’s modified Eagle’s medium (DMEM) (Gibco) supplemented with 10% FBS and 10% PSG. All cells were maintained at 37 °C with 5% CO_2_ in a constant temperature incubator. When the cells reached 80 to 90% confluence, they were subcultured. First, the medium was removed and washed twice with phosphate-buffered saline (PBS). Then, 0.5% trypsin-EDTA (Gibco) was added and incubated at 37 °C for 5 min. Add medium, transfer the suspension to centrifuge tubes and centrifuge at 1000 rpm for 5 min. After centrifugation, discard the supernatant, add medium and pipette. Seed cells into a new dish and maintain at 37 °C with 5% CO_2_.

### Plasmid extraction and lentivirus production

Pelleted bacterial cells were obtained from Academia Sinica (Taipei, Taiwan). The plasmid was extracted by using a Plasmid Mini Kit (Biotools, Taiwan) in accordance with the manufacturer’s protocol.

HEK293T cells were seeded into 10 cm dishes and allowed to attach. In the next day, the cell density should be about 50% confluence for transfection with jetPRIME® in vitro DNA & siRNA transfection reagent (Polyplus, France). The DNA transfection was performed by using a jetPRIME® in vitro DNA & siRNA transfection reagent in accordance with the manufacturer’s protocol. At 24 and 48 h after transfection, replace the transfection medium with 5 mL bovine serum albumin (BSA) (Bio Basic, Canada)-containing medium per plate, and incubate cells for 24 h. Harvest medium- containing lentivirus at 48- and 96-hours post-transfection.

### shRNA infection

Prior to infection, BT-483 and T-47D cells were seeded in 10 cm dishes and allowed to attach for 24 h. Cell density should be approximately 50% confluence for infection. Hexadimethrine bromide (Polybrene) (Bioman, Taiwan) and ESR1 shRNA were added to the medium for 48 h. At 48 h after infection, the medium containing polybrene and ESR1 shRNA was replaced with fresh medium and puromycin (InvivoGen, USA) was added for selection.

### X-ray radiation

X-rays were generated with a small animal irradiator (dose rate, 2.9 Gy/min; RS2000, Rad Source, USA). Cells were placed on a platform and irradiated. Cells were collected 4 and 24 h after irradiation.

### Hydrogen peroxide treatment

Cells were seeded and allowed to adhere for 24 h. After 24 h of incubation, the cells were washed twice with PBS, replaced with fresh medium containing hydrogen peroxide (H_2_O_2_) at a final concentration of 200 µM, and incubated for 4 h. After 4 h of incubation, the cells were collected.

### 3-methyladenine treatment

Cells were seeded and allowed to adhere for 24 h. After 24 h of incubation, the cells were washed twice with PBS, replaced with fresh medium containing 3-methyladenine (3-MA) at a final concentration of 2 mM, and incubated for 0.5 h. After 0.5 h incubation, cells were treated with or without H_2_O_2_ treatment.

### Clonogenic survival assay

Cells were seeded in 10 cm dishes and allowed to attach prior to irradiation. When the cell density was more than 80% confluence, the cells were cultured in a typical manner and seeded into T25 flasks. They were then exposed to X-rays (dose: 0, 2, 4, 6, 8 Gy) and resuspended in 6 cm dishes. The number of cells per dish initially plated increased with increasing dose. After 14 days, they were fixed in 75% methanol and 25% acetic acid and stained with crystal violet (Sigma, USA). Colonies containing more than 50 cells were counted by microscopy and the survival fraction was calculated. Finally, the plating efficiency (PE) and survival fraction were calculated and the survival curve was plotted.

$${{Plating\; efficiency}}\left({{PE}}\right)=\frac{{{Number\; of\; colonies\; counted}}}{{{Number\; of\; cells\; plated}}}* {{100}}$$

$${{Survival\; fraction}}=\frac{{{Number\; of\; colonies\; counted}}}{{{Number\; of\; cells\; plated}}* {{PE}}}* {{100}}$$

### RT-PCR Analysis

RNA was extracted by using a RNeasy Kit (Qiagen, Germany) in accordance with the manufacturer’s protocol. cDNA reverse transcription was performed by using a Quant II Fast RT Kit (Biotools, Taiwan) in accordance with the manufacturer’s protocol. The cDNA was diluted to 50 ng/μL and we used the SYBR qPCR Mix (Biotools, Taiwan) for real-time quantitative detection. We performed a total of 40 cycles in a reaction volume of 20 μL. The amplification protocol consisted of the following steps: predenaturation at 95 °C for 10 min, denaturation at 95 °C for 15 s, annealing at 60 °C for 20 s, and primer template extension at 72 °C for 15 s. We used the StepOne PCR system software (Allied Business Intelligence Inc., USA) to complete the data collection. The CT values were calibrated with S26 as an internal control and the target gene expression was analyzed using the 2(-ΔCT) method. The following are primers and sequences.

### Western blot analysis

Cell pellets were lysed with RIPA buffer (Thermo, USA) containing a proteinase (Thermo) and phosphatase inhibitor cocktail (Thermo). Cell lysate was centrifugated at 15,000 g for 30 min at 4 °C. The protein concentration was determined using a BCA Protein Assay Kit (Thermo). To denature the protein, it was subjected to a temperature of 100 °C in a water bath for a duration of 10 min. Subsequently, we used SDS-polyacrylamide gel electrophoresis (SDS-PAGE) to analyze the protein. The voltage for protein concentration was set at 80 V, while for the separation step, it was increased to 100 V. The polyvinylidene fluoride membranes (Merck, USA) were transferred at 0.4 A for 90 min. The membrane was blocked with 5% nonfat milk for 1 h and incubated with the primary antibodies overnight at 4 °C. The next day, after washing with Tris buffered saline with 2% Tween 20 (TBST), the membranes were incubated with the appropriate secondary antibody for 1 h at 4 °C. The images were captured using an electro-chemiluminescence immunoassay method. Subsequently, the obtained images were analyzed using Image J software. The following is the information regarding antibodies.

**Table:** 

Antibody	Catalog number	Company	Dilution
Estrogen receptor α	8644S	Cell Signaling	1:1000
Phospho-ESR1 (Ser118)	ab32396	Abcam	1:1000
Phospho-ESR1 (Ser106)	ab75753	Abcam	1:1000
Phospho-ATM (Ser1981)	ab81292	Abcam	1:20000
ATM	ab32420	Abcam	1:5000
Ku70	A7330	ABclonal	1:1000
XRCC5/Ku80	16389-1-AP	Proteintech	1:1000
XRCC4	GTX109632	Genetex	1:1000
BRCA1	GTX100720	Genetex	1:2000
Rad51	GTX100108	Genetex	1:5000
Bcl-2	A0208	ABclonal	1:1000
Caspase 9/p35/p10	10380-1-AP	Proteintech	1:1000
Caspase 3/p17/p19	19677-1-AP	Proteintech	1:1000
LC3B	GTX127375	Genetex	1:1000
COX2	A2413	ABclonal	1:1000
GPX4	A1933	ABclonal	1:1000
Phospho-IRE1-α (Ser724)	PA1-16927	ThermoFisher	1:1000
P62	ab207305	Abcam	1:1000
Phospho-p62 (Ser349)	95697	Cell signaling	1:1000
P53	ab32389	Abcam	1:1000
P21	ab188224	Abcam	1:1000
PUMA	ab9643	Abcam	1:1000
β-actin	66009-1-Ig	Proteintech	1:5000

### Immunofluorescence

Cells were seeded at a density of 4–5*10^5^ cells/well for 24 h and treated with hydrogen peroxide for 4 h. After incubation for 4 h, the cells were fixed with 4% paraformaldehyde for 10 min at 37 °C and 70 rpm, washed with methanol for 10 min at room temperature, permeabilized with 0.1% Triton X-100 for 15 min at room temperature, and blocked with 5% BSA for 90 min at 37 °C and 70 rpm. Cells were incubated with primary antibodies overnight at 4 °C. The next day, after washing with phosphate-buffered saline (PBS), the cells were incubated with Alexa Fluor 488 secondary antibody (#A-11008; Invitrogen, USA) or Alexa Fluor 594 secondary antibody (#A-11005; Invitrogen, USA) for 90 min at 37 °C and 70 rpm. The cells were washed and incubated with Hoechst 33342 for 45 min to stain the nuclei. We added 5 μL of ProLong™ Gold Antifade Mountant (Invitrogen) to protect the fluorescent dyes from fading. Finally, images were captured using a Zeiss LSM880 confocal microscope.

### Animal model

All animal experiments were approved by the Institutional Animal Care and Use Committee (IACUC) of National Yang Ming Chiao Tung University (approval no. 1131112) and were conducted in accordance with institutional guidelines. Age-matched female mice with a severe combined immunodeficiency mutation (NOD/SCID) (6–8 weeks old) originally from The Jackson Laboratory were used. To estimate in vivo tumor growth, 5 × 10⁶ cells were resuspended in 0.1 mL of PBS and injected subcutaneously into the backs of the mice. Tumor volumes were measured weekly. Tumor masses were harvested after three to six weeks. The animal groups were neither blinded nor randomized. However, statistically significant differences in tumor growth size were observed among the various cell groups (*n* = 6 per group).

### Statistical analysis

Data were presented as mean ± standard deviation (SD). We used GraphPad Prism (version 9) (GraphPad, USA) to perform Student’s t-test to determine statistical significance. Statistical significance was evaluated by Student’s t-test at *, *p* < 0.05; **, *p* < 0.01; ***, *p* < 0.005 to represent differences between groups.

## Supplementary information

Revised supplementary information

Related Manuscript File
