# Supplementary material for: Targeting ESR1 restores SQSTM1-dependent autophagy and sensitizes ER-positive breast cancer to oxidative and radiation stress
Source: Cell Death Discov. 2025 Oct 7;11:451. doi: 10.1038/s41420-025-02755-8 (PMC12504691; doi:10.1038/s41420-025-02755-8)

**Supplementary information**

**Targeting ESR1 restores SQSTM1-dependent autophagy and sensitizes ER-positive breast cancer to oxidative and radiation stress**

Yi-Fang Yang^1^, Zhao-Jing He^2^, Han-Hsi Kuo^2^, You-Yu Lin^2^, Cheorl-Ho Kim^3^, Huei-Yu Cai^2^, Chi-Long Chen^4^, Michael Hsiao^5^, Ying-Chung Chen^6^, Peter Mu-Hsin Chang^7, 8*^, Yu-Chan Chang^2*^

1. Department of Medical Education and Research, Kaohsiung Veterans General Hospital, Kaohsiung, Taiwan.
2. Department of Biomedical Imaging and Radiological Sciences, National Yang Ming Chiao Tung University, Taipei, Taiwan
3. Molecular and Cellular Glycobiology Unit, Department of Biological Sciences, SungKyunKwan University, Suwon, Gyunggi-Do 16419, Republic of Korea
4. Department of Pathology, College of Medicine, Taipei Medical University and Taipei Medical University Hospital, Taipei, Taiwan
5. Genomics Research Center, Academia Sinica, Taipei, Taiwan
6. Department of Physiology and Biophysics, National Defense Medical Center, Taipei, Taiwan
7. Institute of Biopharmaceutical Sciences, National Yang Ming Chiao Tung University, Taiwan
8. Department of Oncology, Taipei Veterans General Hospital

#To whom correspondence should be addressed:

Dr. Yu-Chan Chang, Department of Biomedical Imaging and Radiological Sciences, National Yang Ming Chiao Tung University, Taipei, Taiwan, Tel: +886-2-2826-7064, E-mail: yuchanchang@nycu.edu.tw.

&

Dr. Peter Mu-Hsin Chang, Institute of Biopharmaceutical Sciences, National Yang Ming Chiao Tung University, Taiwan. Tel: +886-2-28757762, E-mail: ptchang@vghtpe.gov.tw

**Competing Interests**

No conflicts of interest were declared.

**Running Title:** ESR1 compromise the effectiveness of radiotherapy effect.

**Supplementary figure legends**

**Supplementary Figure 1.** RNA expression level of exogenous ESR1 model with or without irradiation exposure. The data from three independent experiments are presented as the means ± SEM.

**
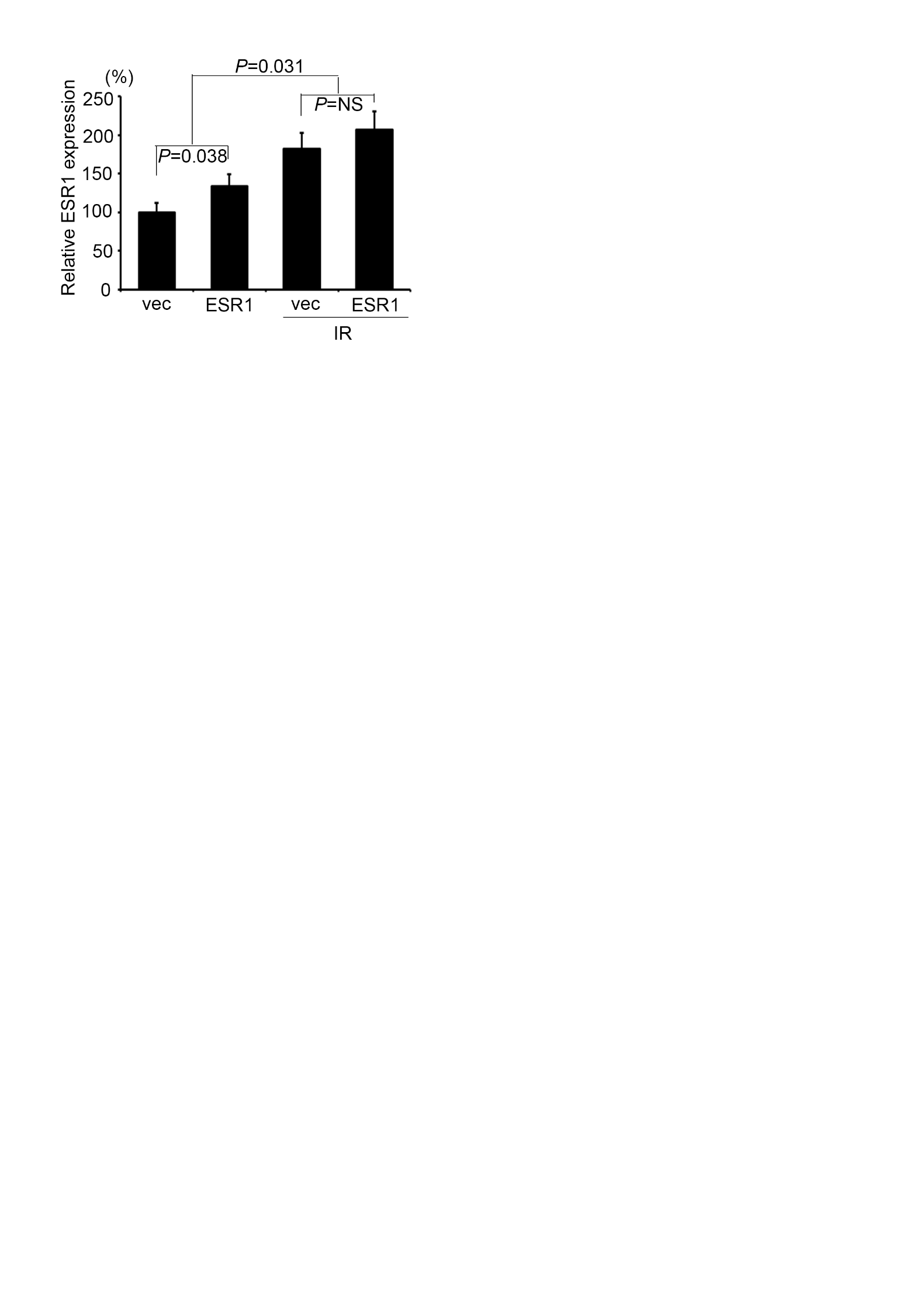
**

**Supplementary Figure 2. (A)** Protein levels of p53, p21, and PUMA in MCF7 cells with or without p53 knockdown. Actin was used as an internal control. **(B)** Survival fraction of the MCF7 model with or without p53 knockdown followed by irradiation. The data from three independent experiments are presented as the means ± SEM.

**
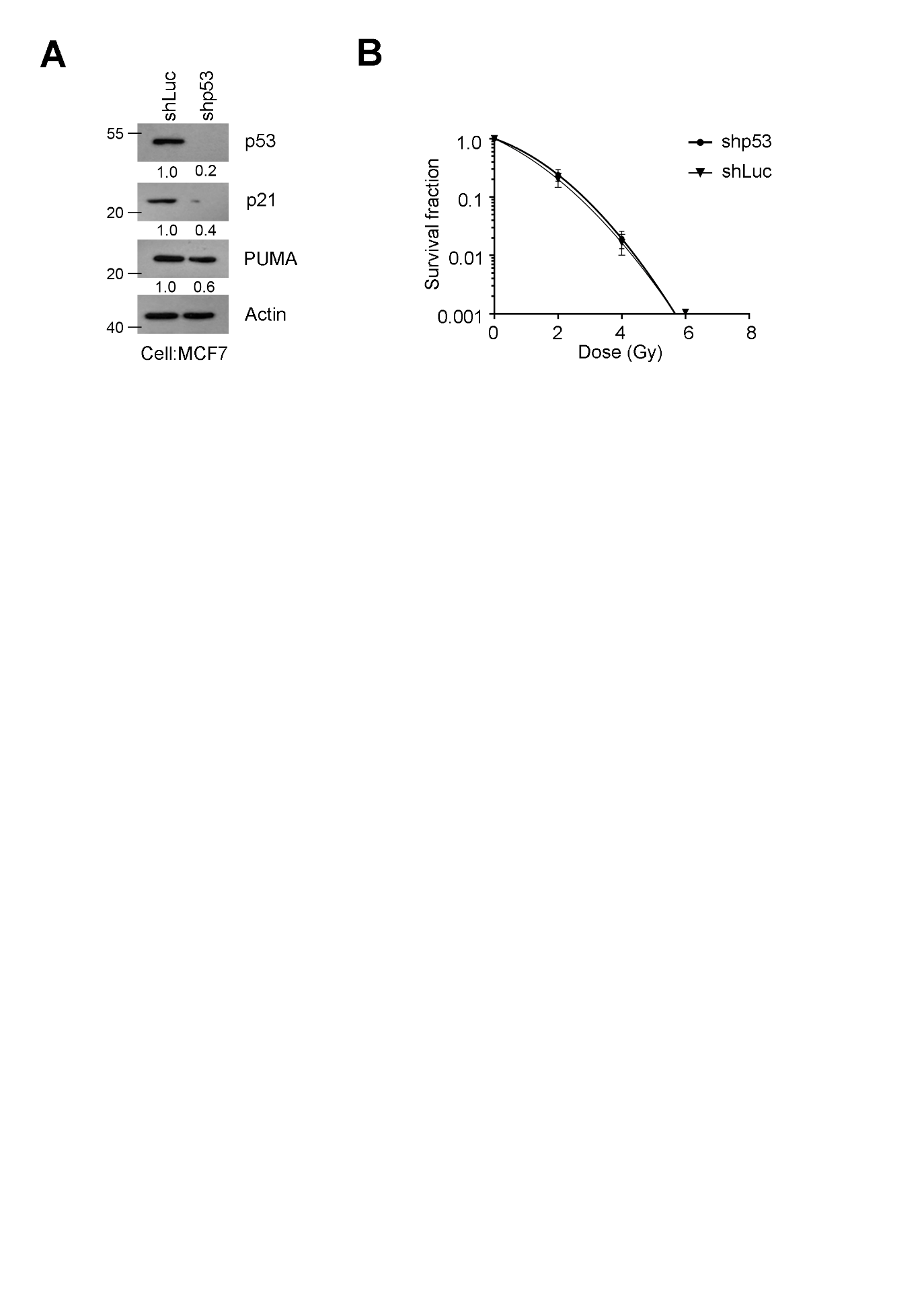
**

**Supplementary Figure 3. (A)** Protein levels of pESR1 (S118) and ESR1 in T-47D cells with or without ESR1 overexpression. Actin was used as an internal control. **(B)** Survival fraction of the T-47D isogenic models after irradiation. The data from three independent experiments are presented as the means ± SEM.


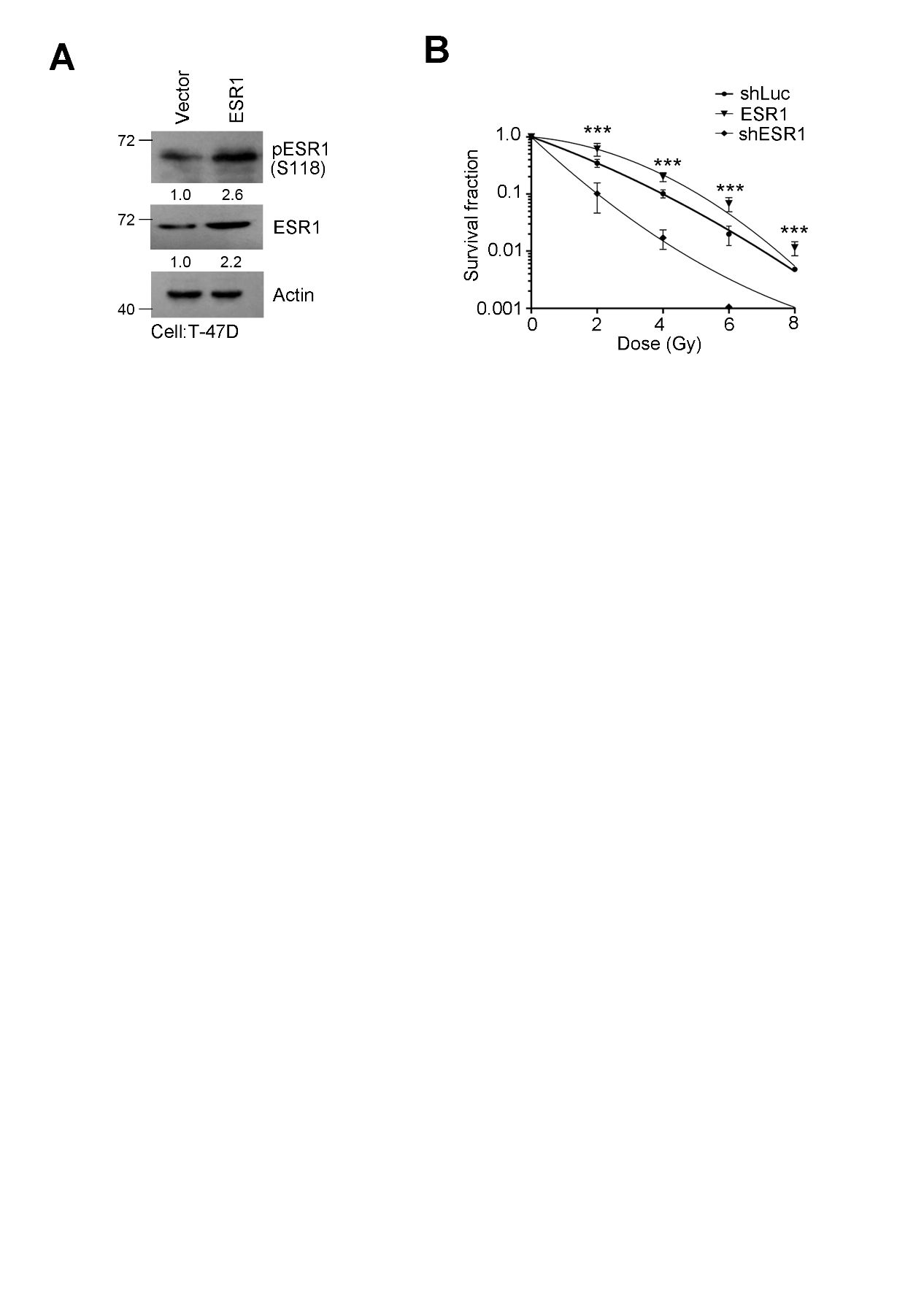


**Supplementary Figure 4.** Protein levels of Bcl-2, caspase 9 (full length/cleaved) and caspase 3 in BT-483 with or without ESR1 knockdown in a radiation dose-dependent manner. Actin was used as an internal control.


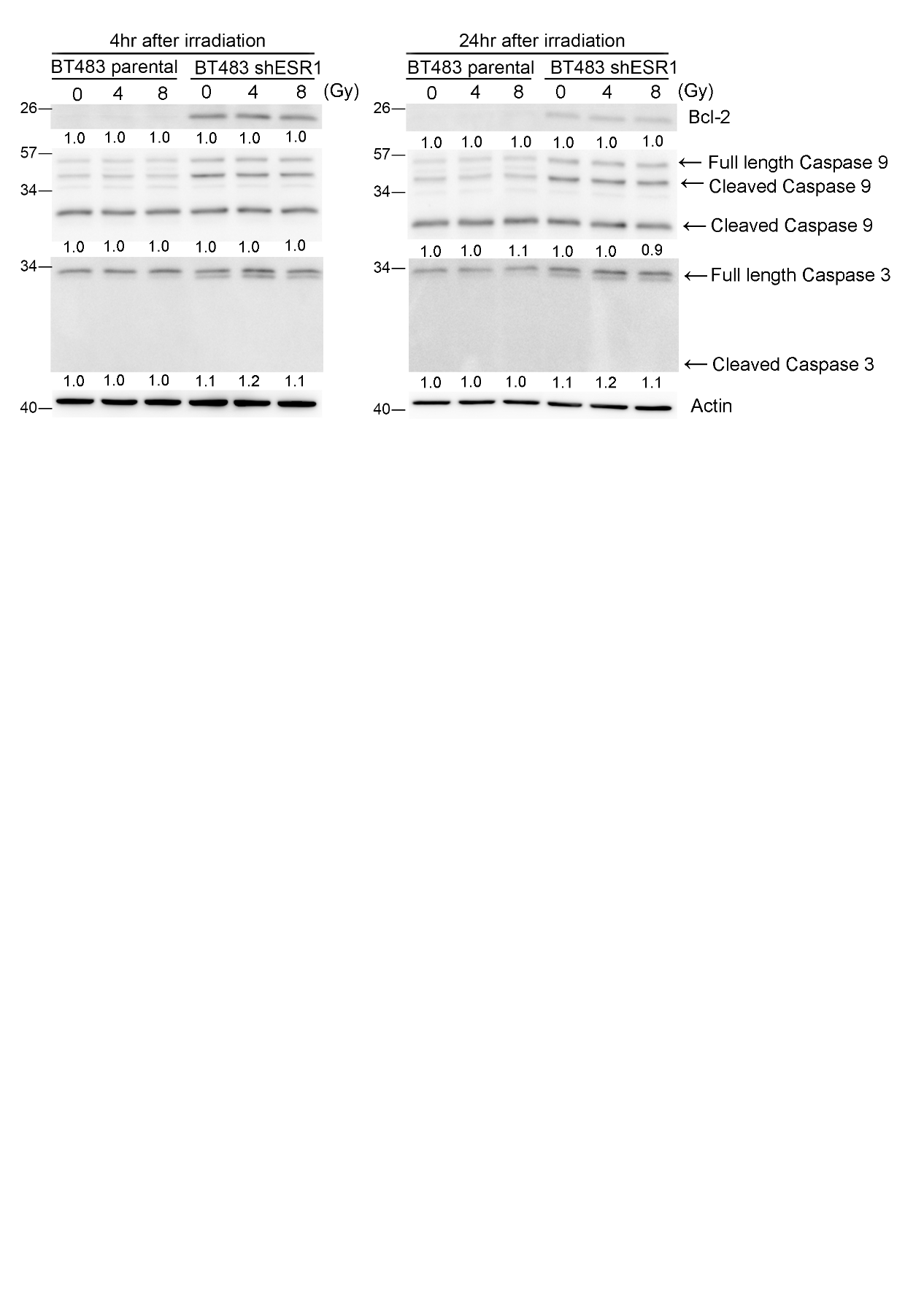


**Supplementary Figure 5.** Protein levels of LC3B (I/II) and p62 in MCF7 with or without ESR1 overexpression in a radiation dose-dependent manner. Actin was used as internal control.

**
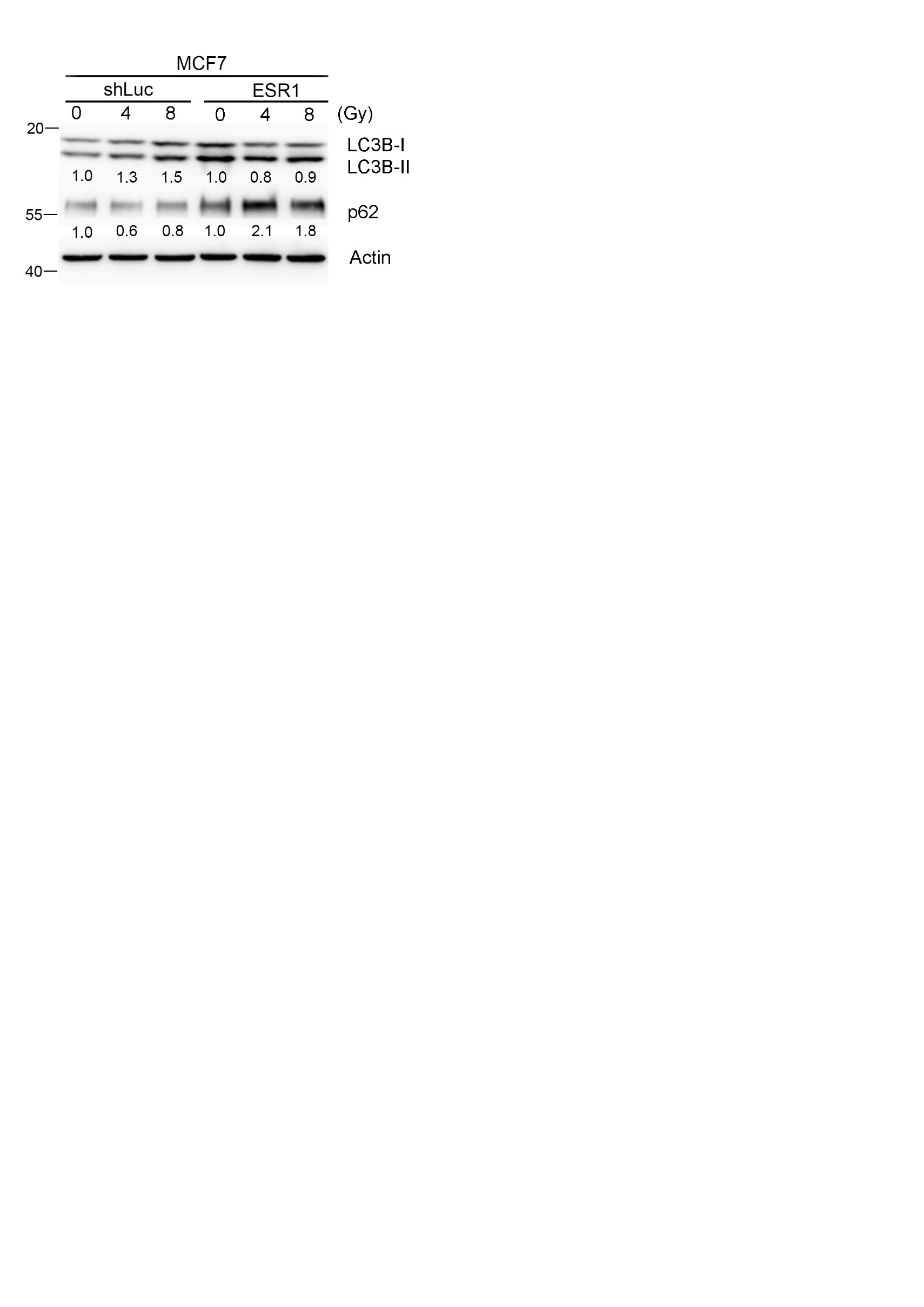
**

**Supplementary Figure 6. (A)** Quantification of LC3B fluorescence signals in the ESR1 overexpression or knockdown models, respectively. **(B)** Quantification of LAMP2 fluorescence signals in the ESR1 overexpression model. The data from three independent experiments are presented as the means ± SEM.

**
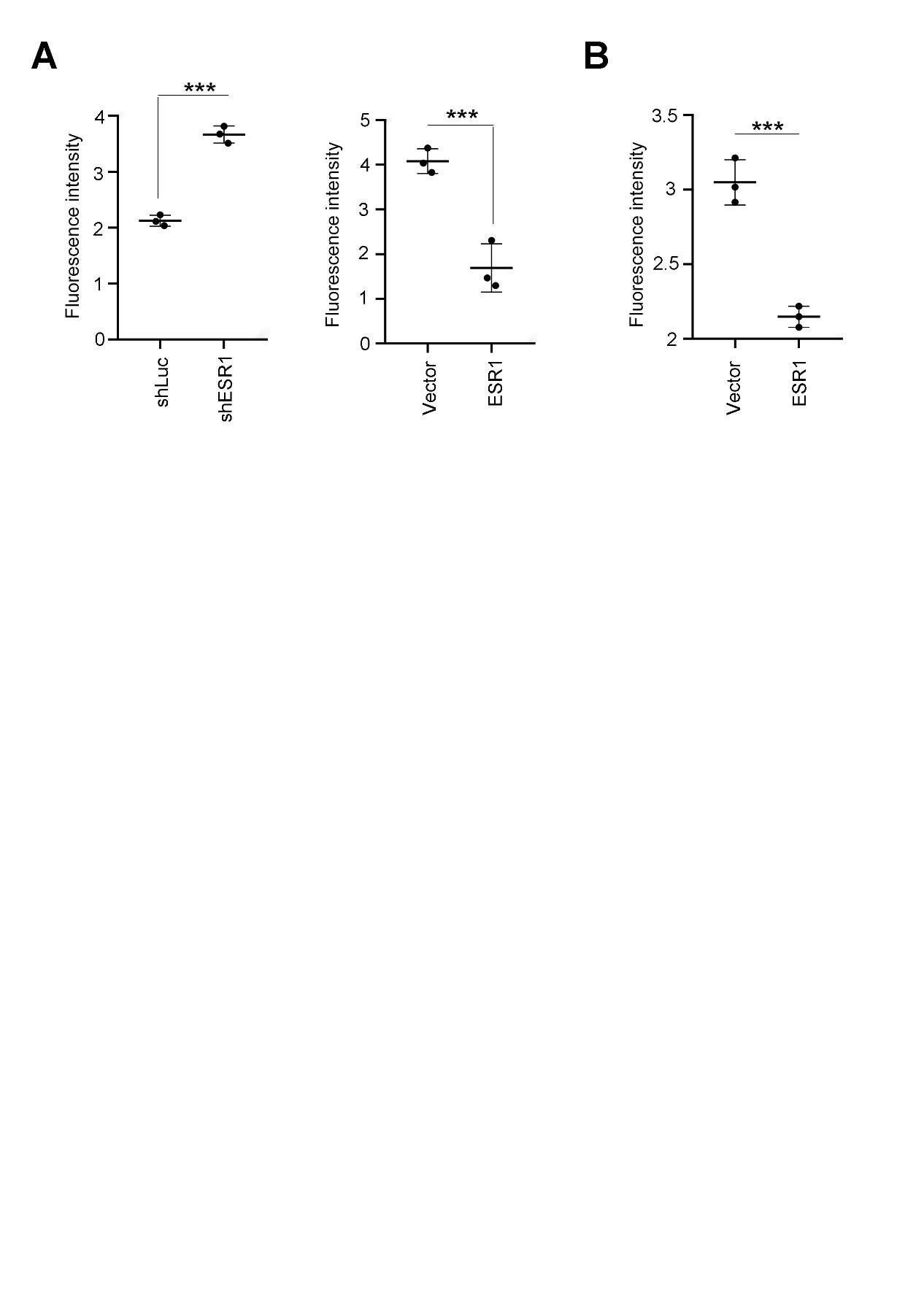
**

**Supplementary Figure 7.** Colony forming ability of T-47D ESR1 knockdown with or without E2 treatment.


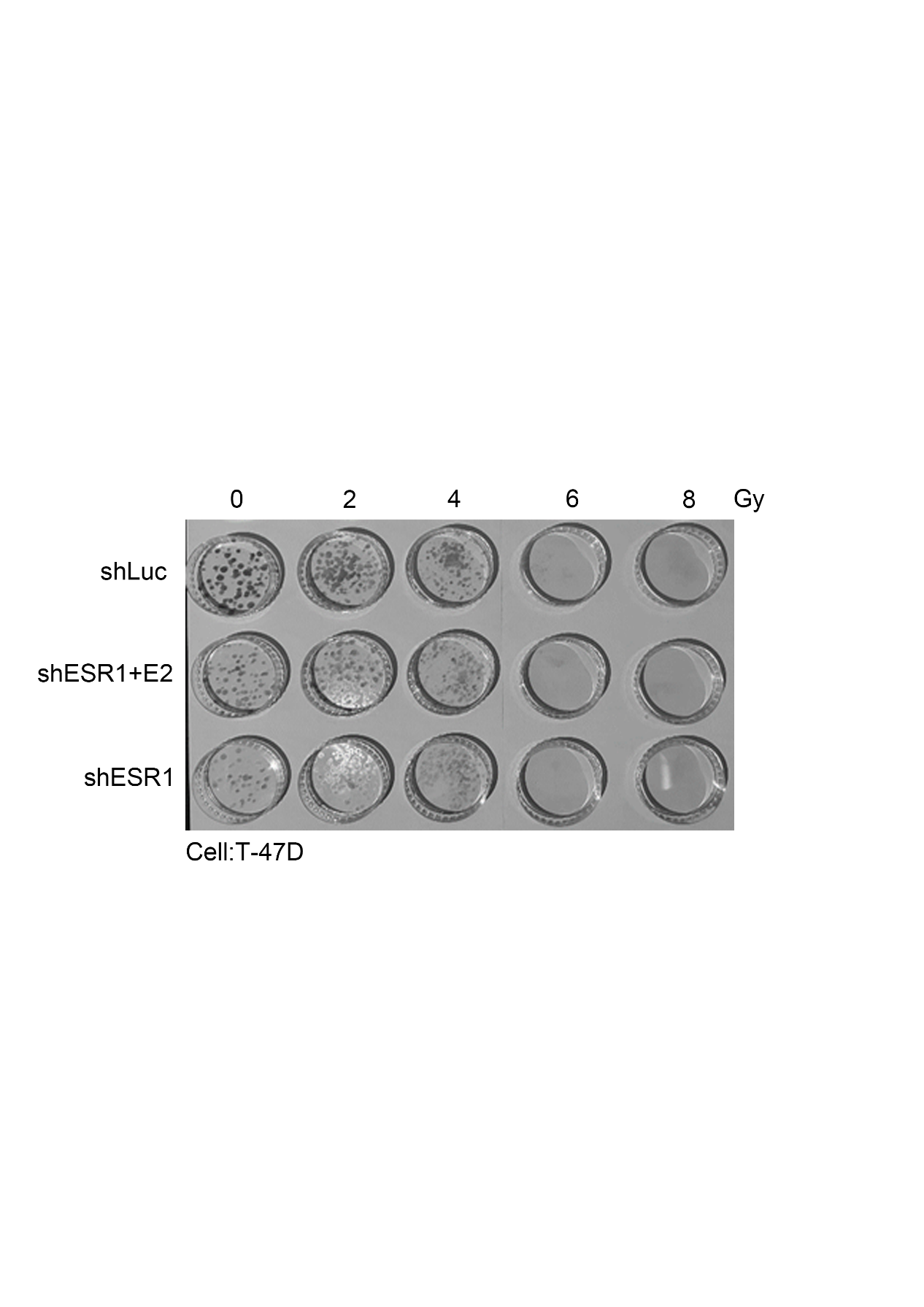


**Supplementary Figure 8.** Heatmap showing ER status, breast cancer molecular classification, ESR1/SQSTM1 expression in breast cancer patients from the TCGA cohort.


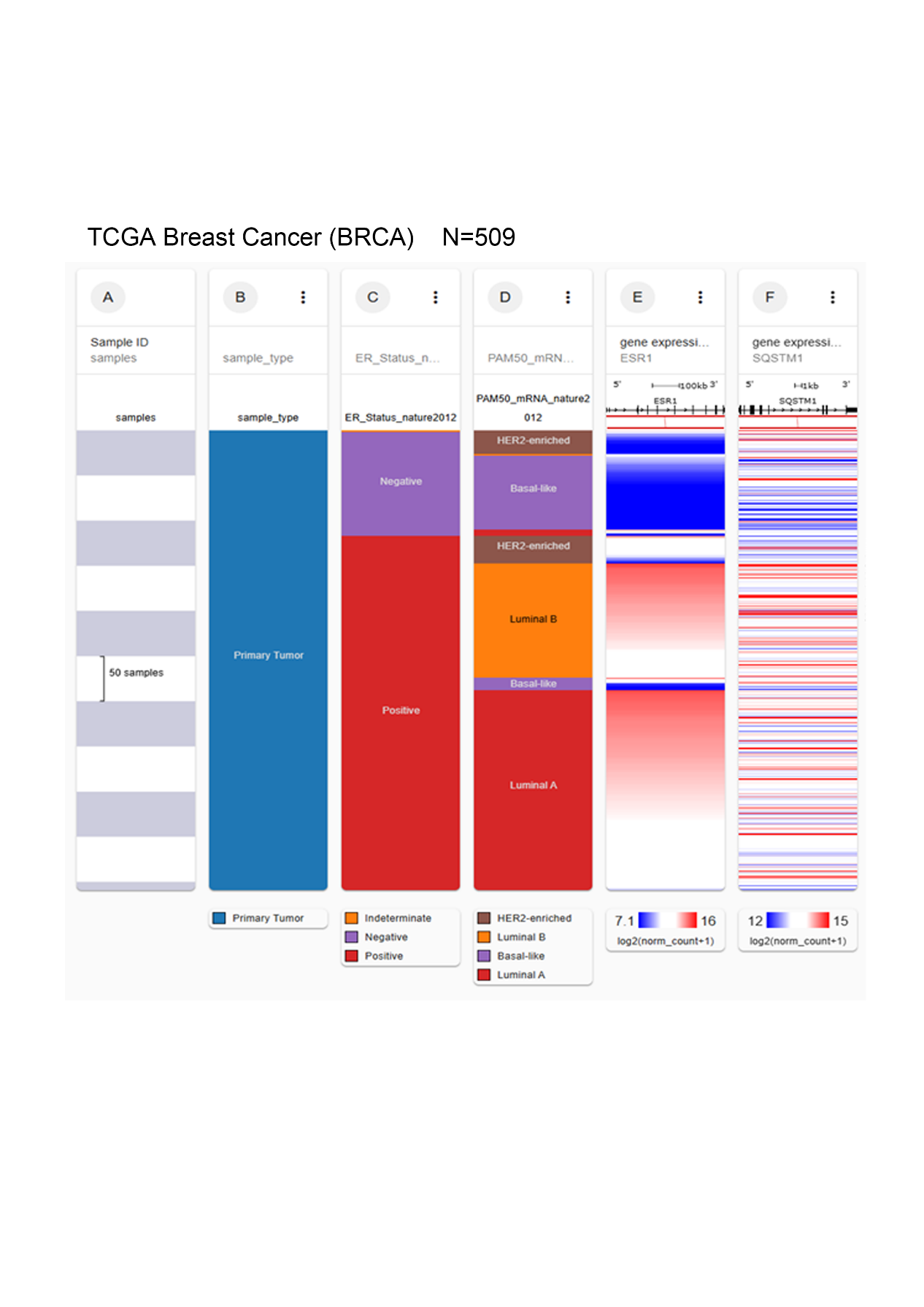


**Supplementary Figure 9.** Correlation between ESR1 and SQSTM1 (p62) expression levels in ER^+^ breast cancer patients from the TCGA cohort.


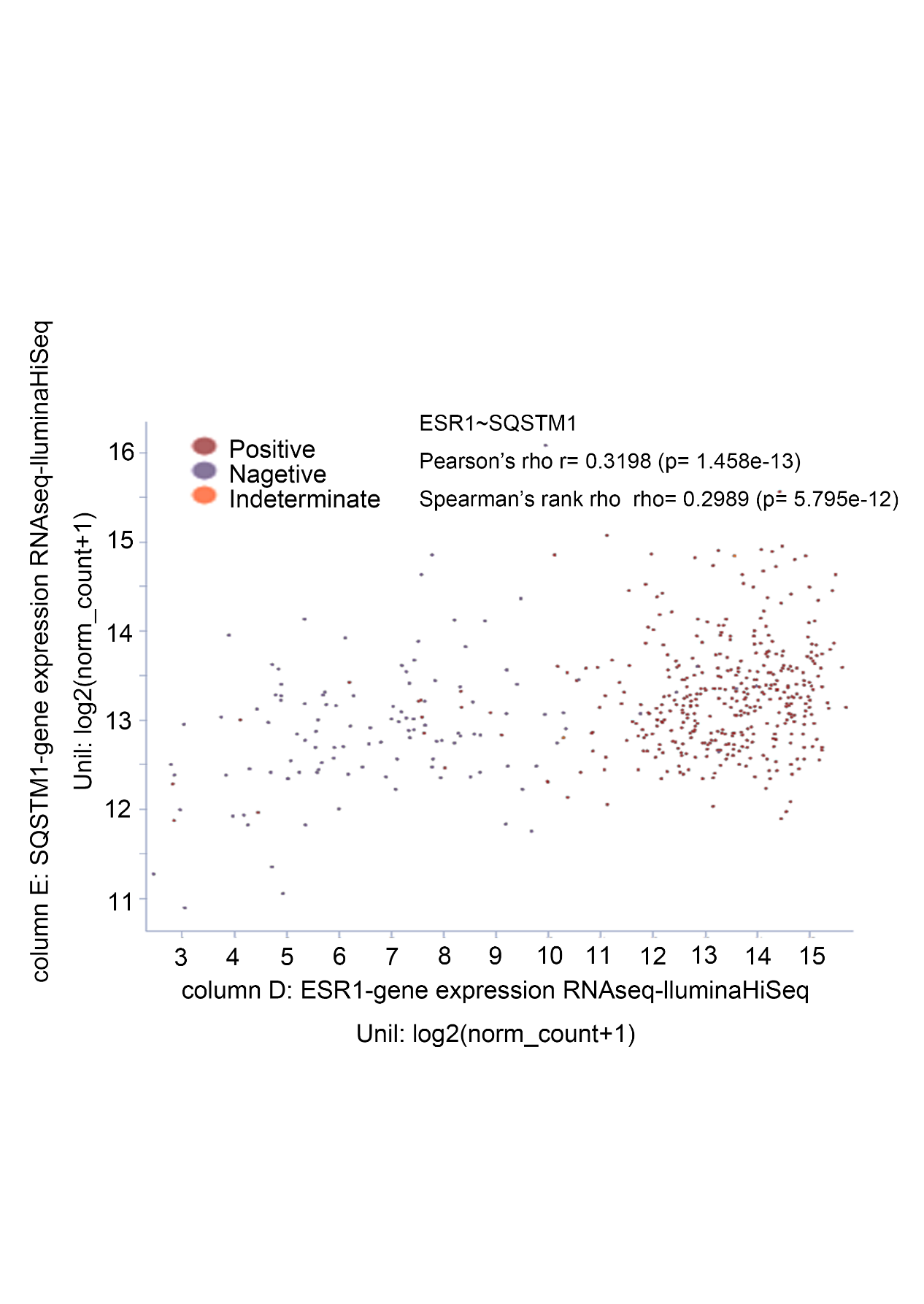

Supplement: Supplementary file 1 — Revised supplementary information [file 41420_2025_2755_MOESM1_ESM.docx]
