# Supplementary material for: Targeting ESR1 restores SQSTM1-dependent autophagy and sensitizes ER-positive breast cancer to oxidative and radiation stress
Source: Cell Death Discov. 2025 Oct 7;11:451. doi: 10.1038/s41420-025-02755-8 (PMC12504691; doi:10.1038/s41420-025-02755-8)

Fig. 2A

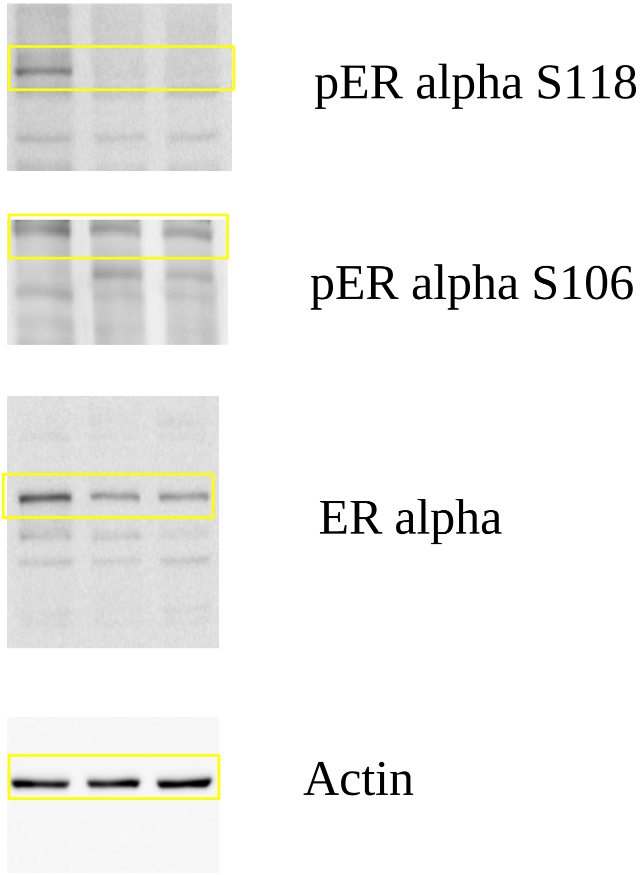
pER alpha S118
pER alpha S106
ER alpha
Actin

Fig. 2B

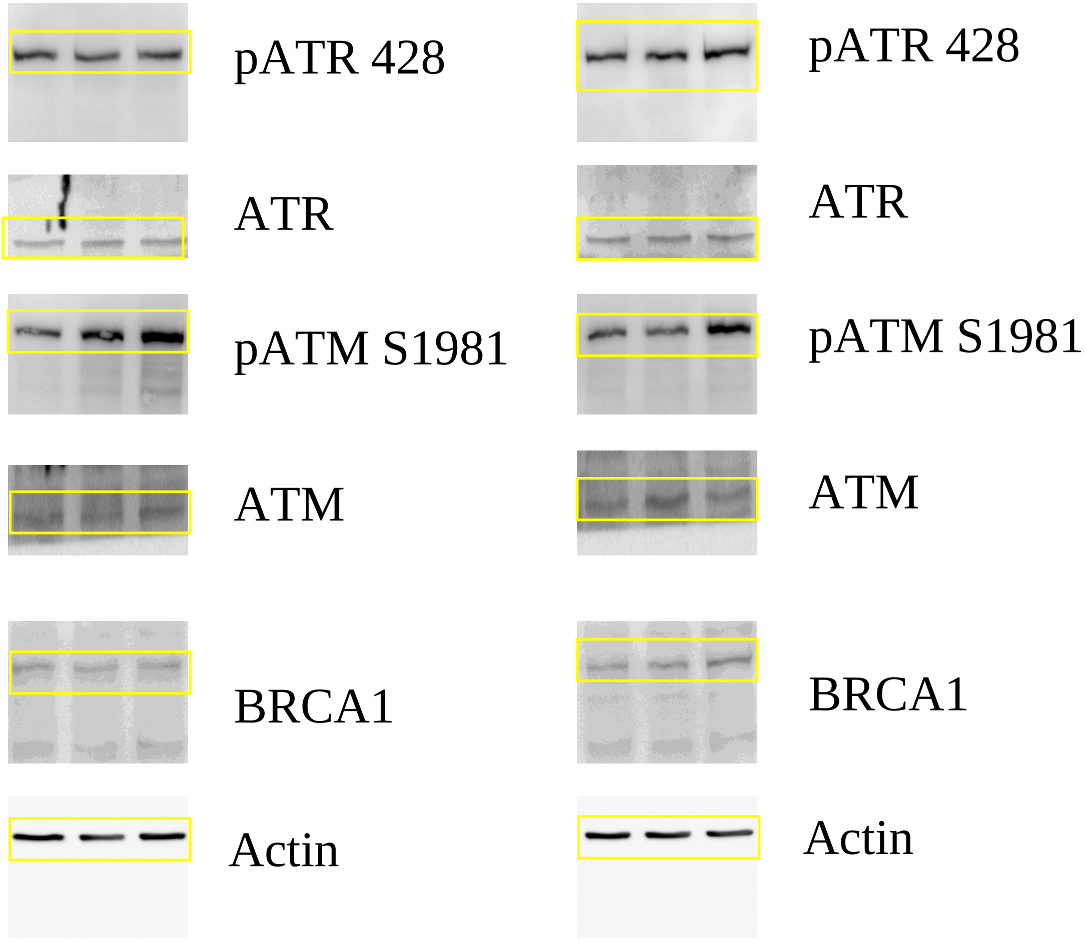
pATR 428
ATR
pATM S1981
ATM
BRCA1
Actin
pATR 428
ATR
pATM S1981
ATM
BRCA1
Actin

Fig. 2H

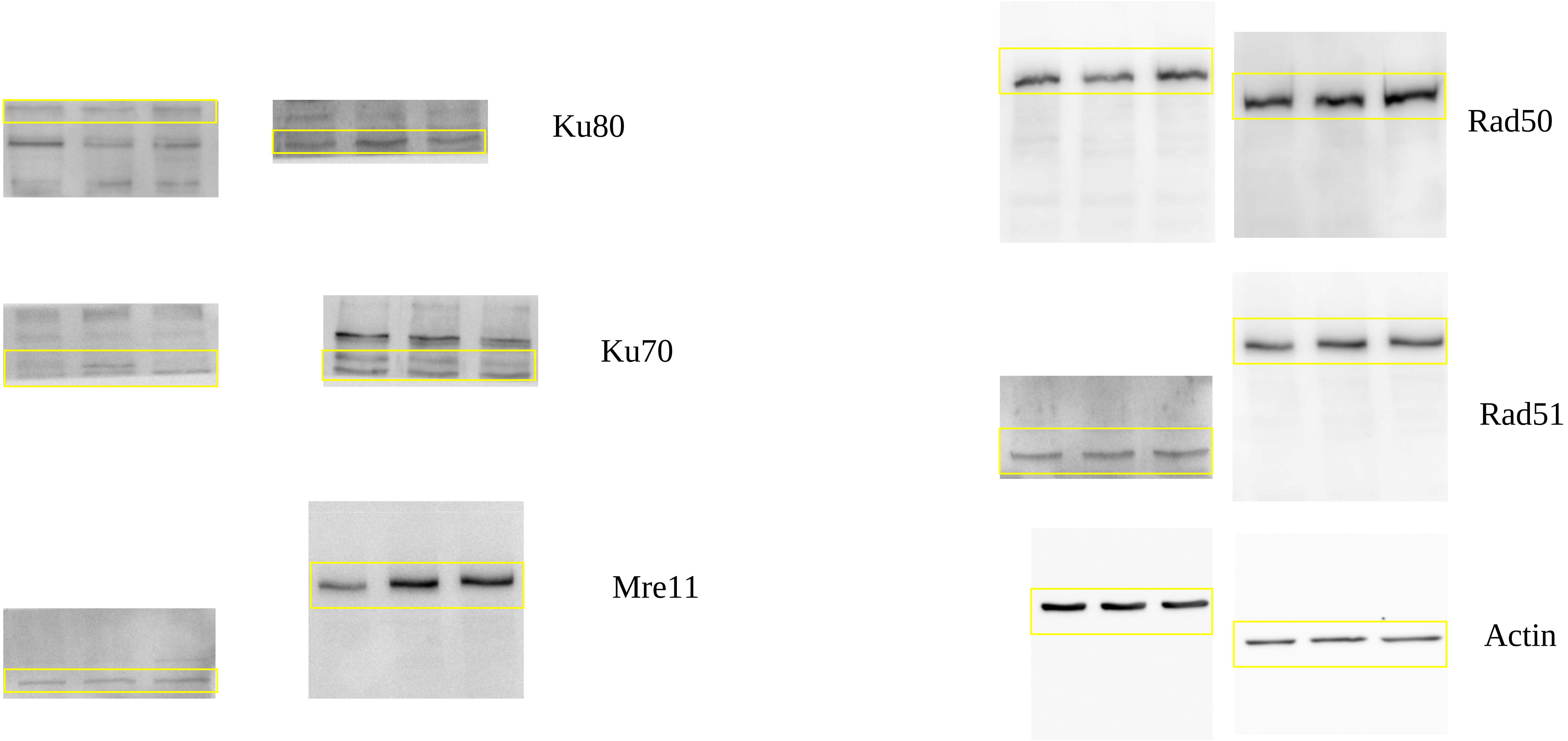

Fig. 3B

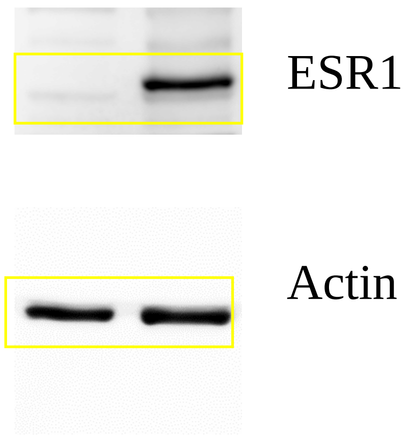

Fig. 3G

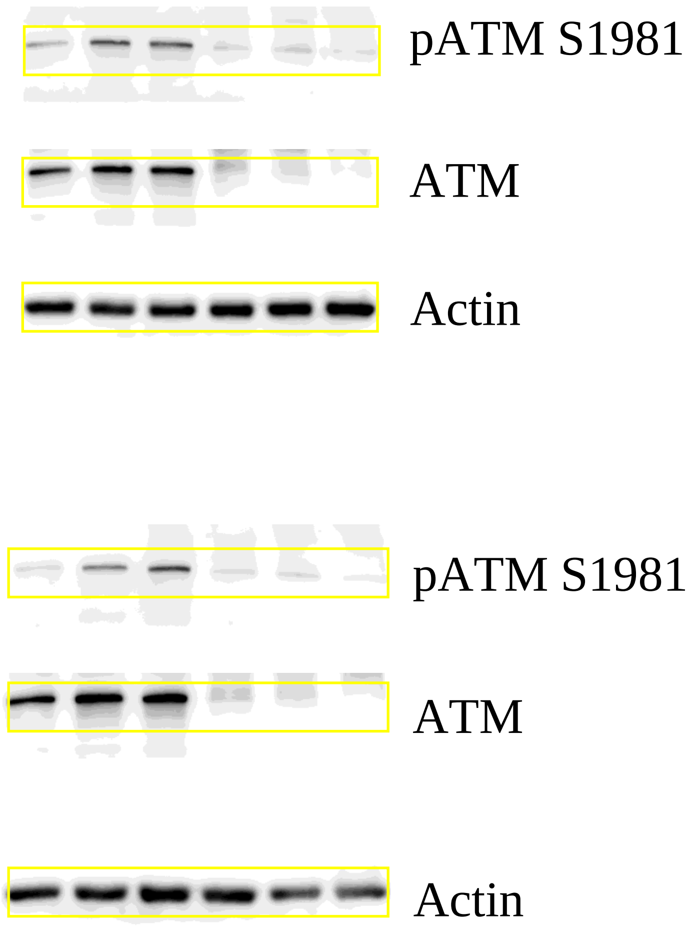

Fig. 3H

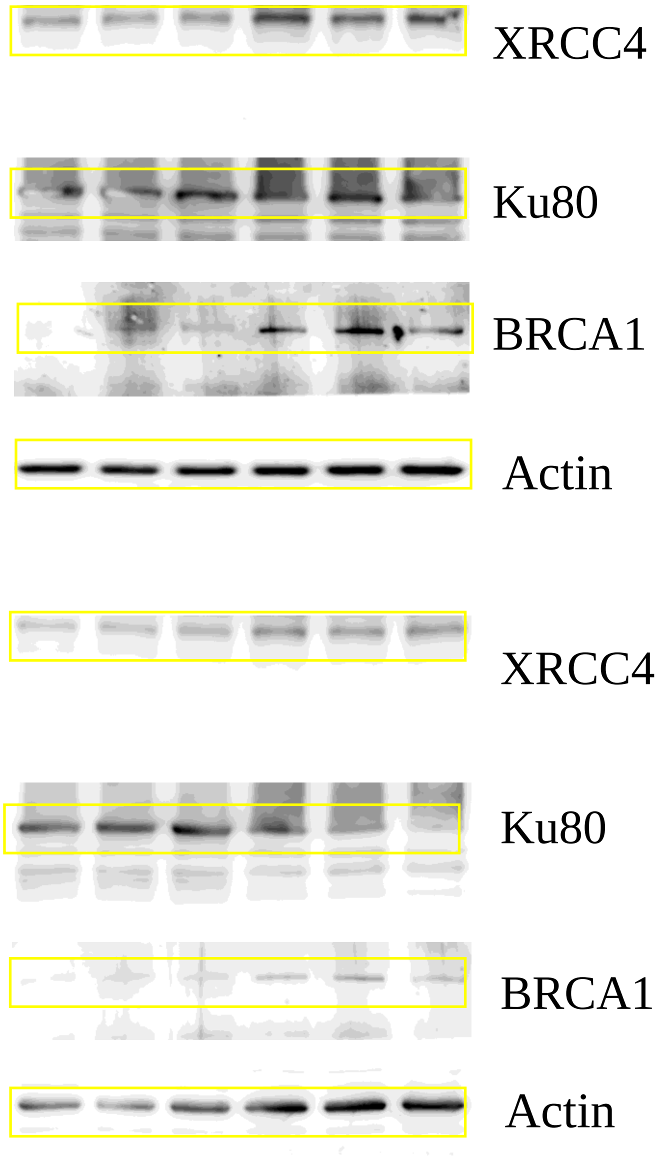

Fig. 4A

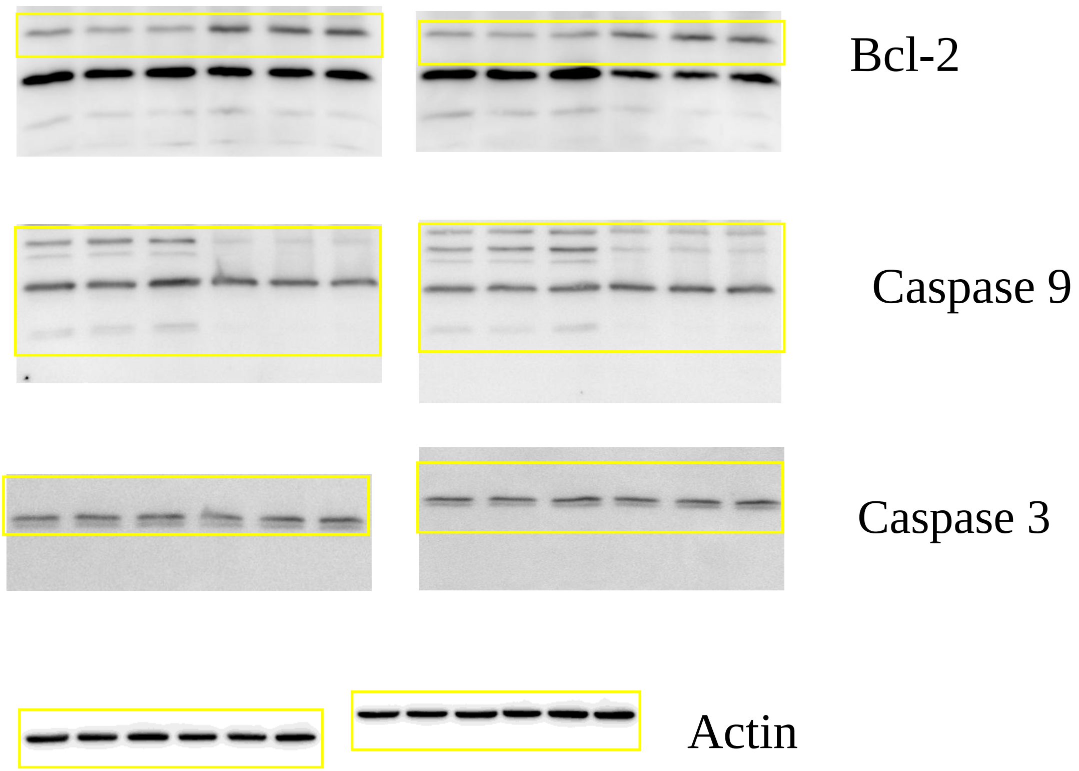

Fig. 4B

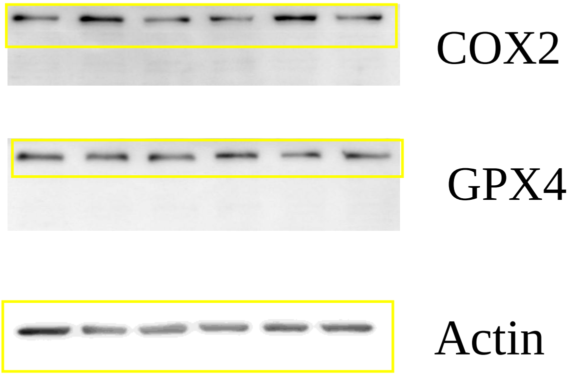

Fig. 4C

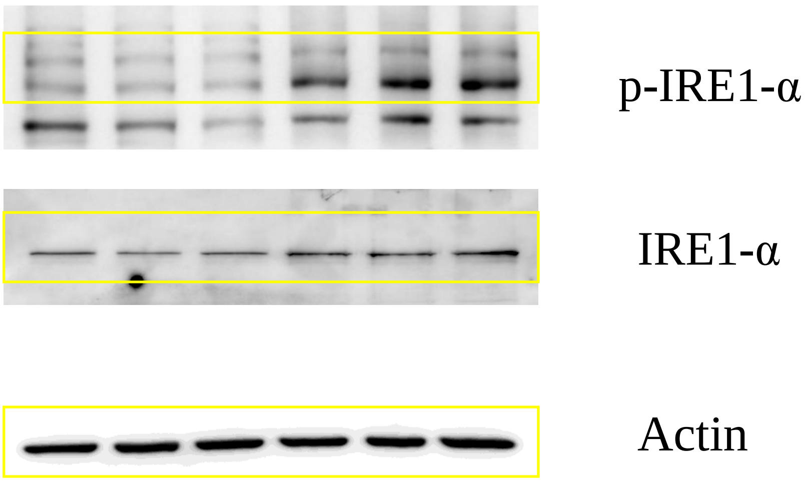

Fig. 4D

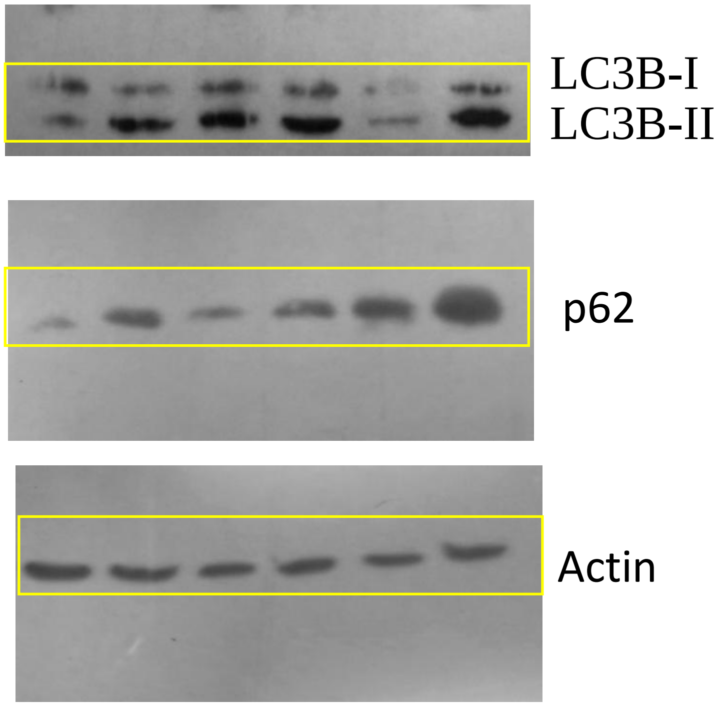

Fig. 4E

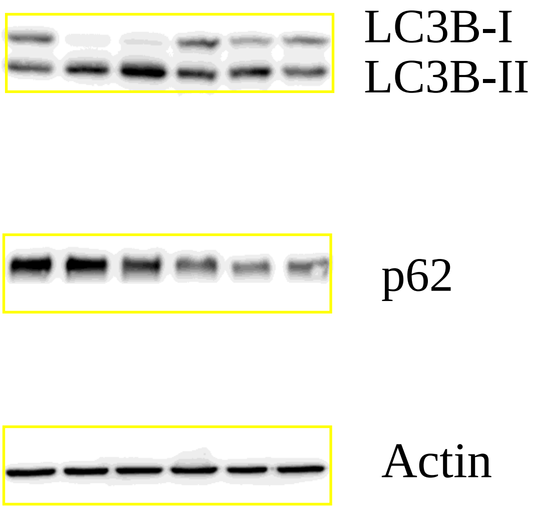

Fig. 5A

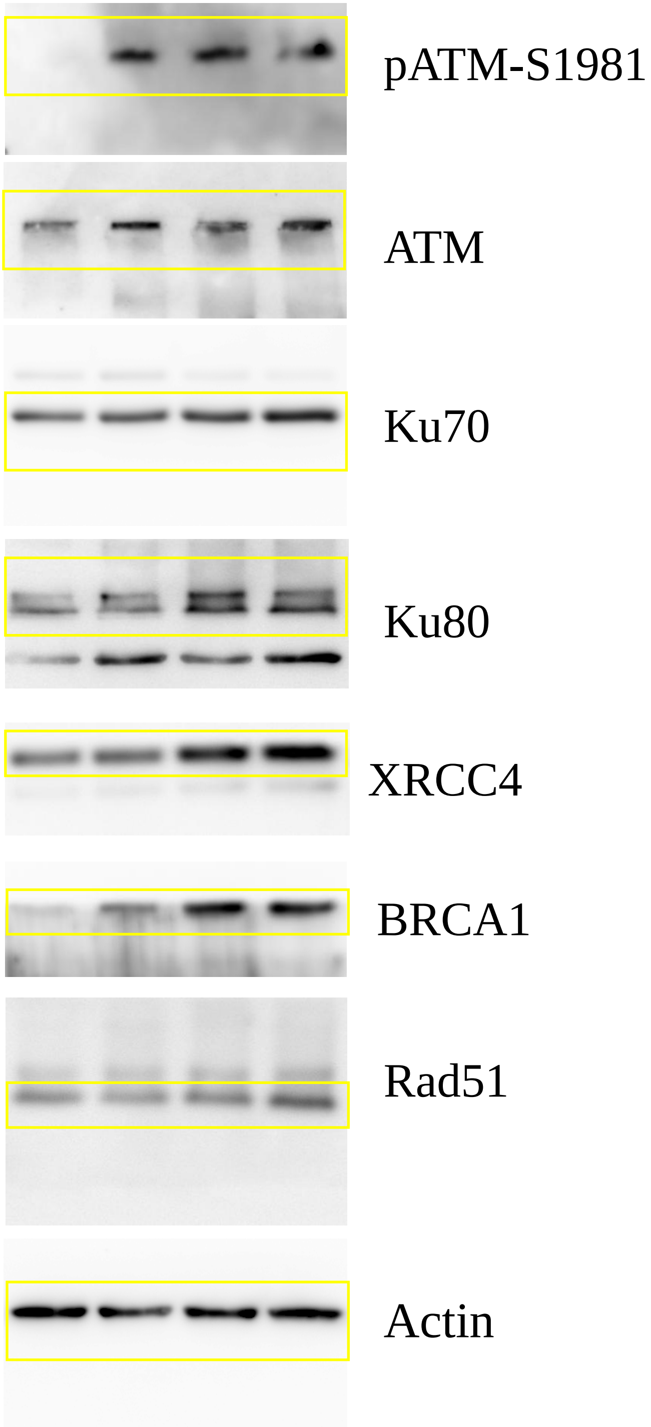
pATM-S1981
ATM
Ku70
Ku80
XRCC4
BRCA1
Rad51
Actin

Fig. 5C

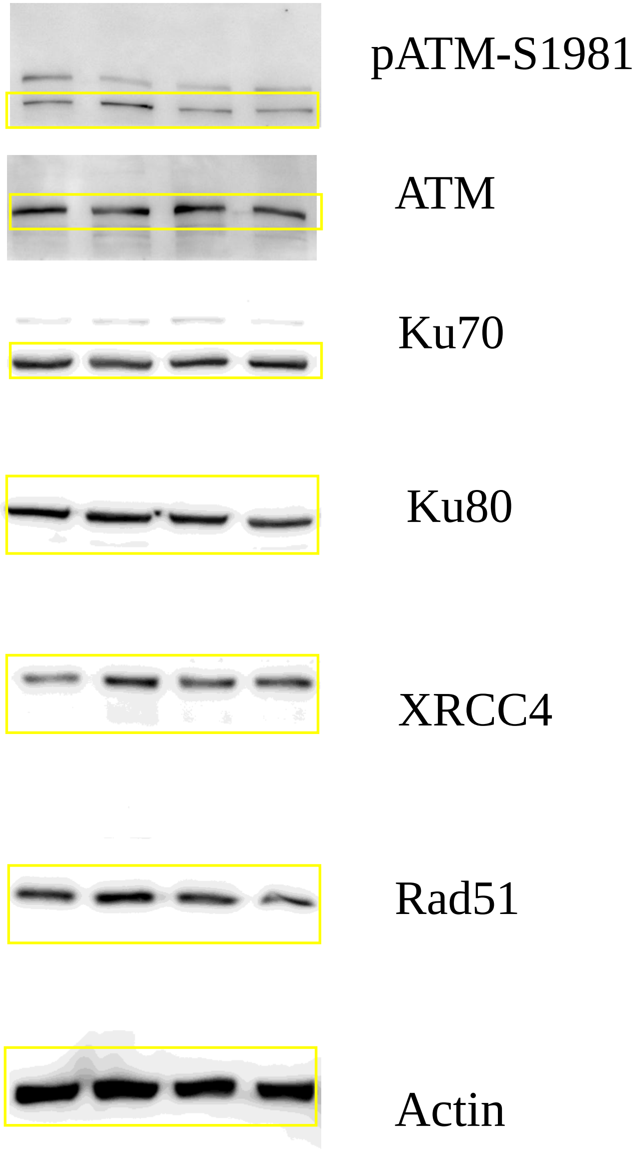
pATM-S1981
ATM
Ku70
Ku80
XRCC4
Rad51
Actin

Fig. 5E

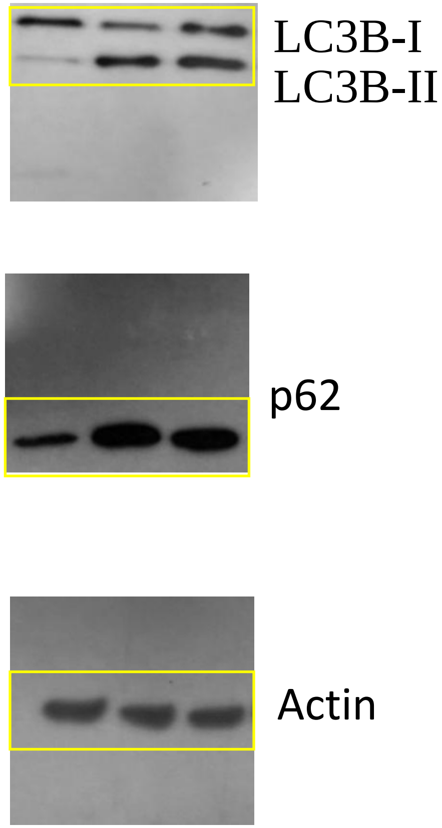

Fig. 5G

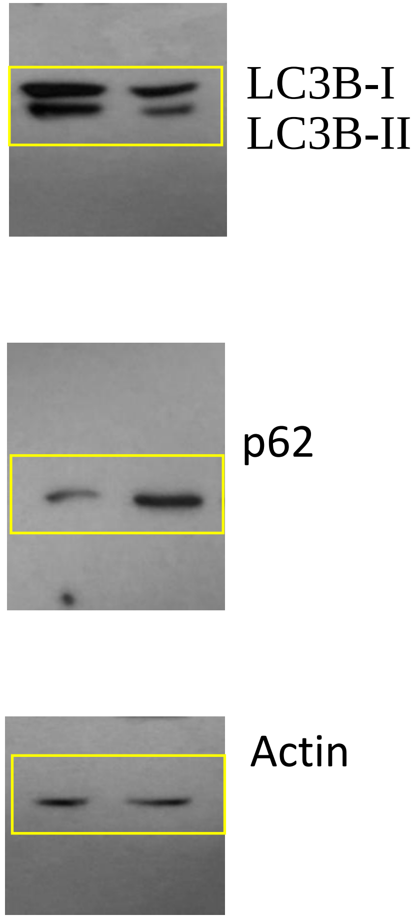

Fig. 6A

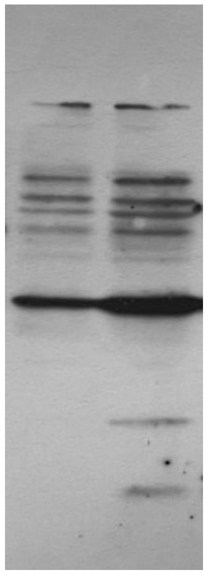

Fig. 6D

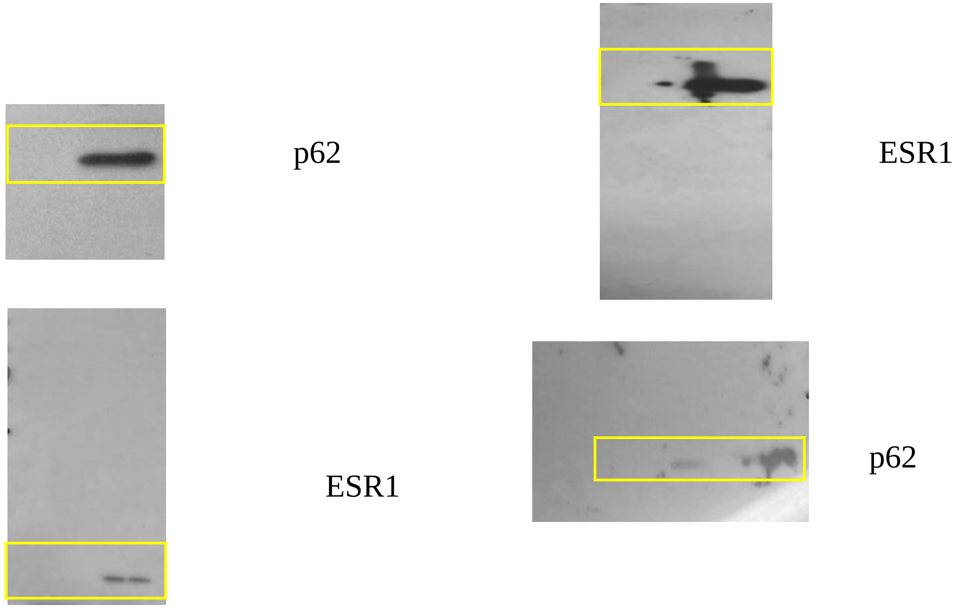

Fig. 6E

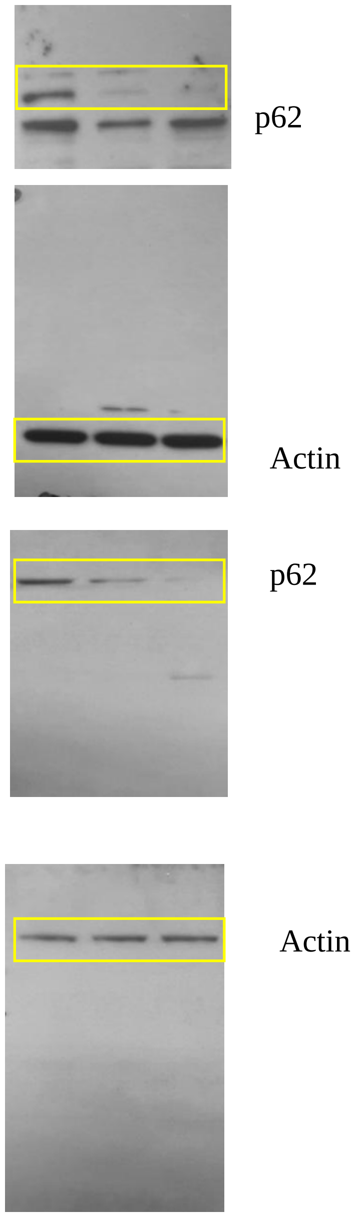

Fig. 6F

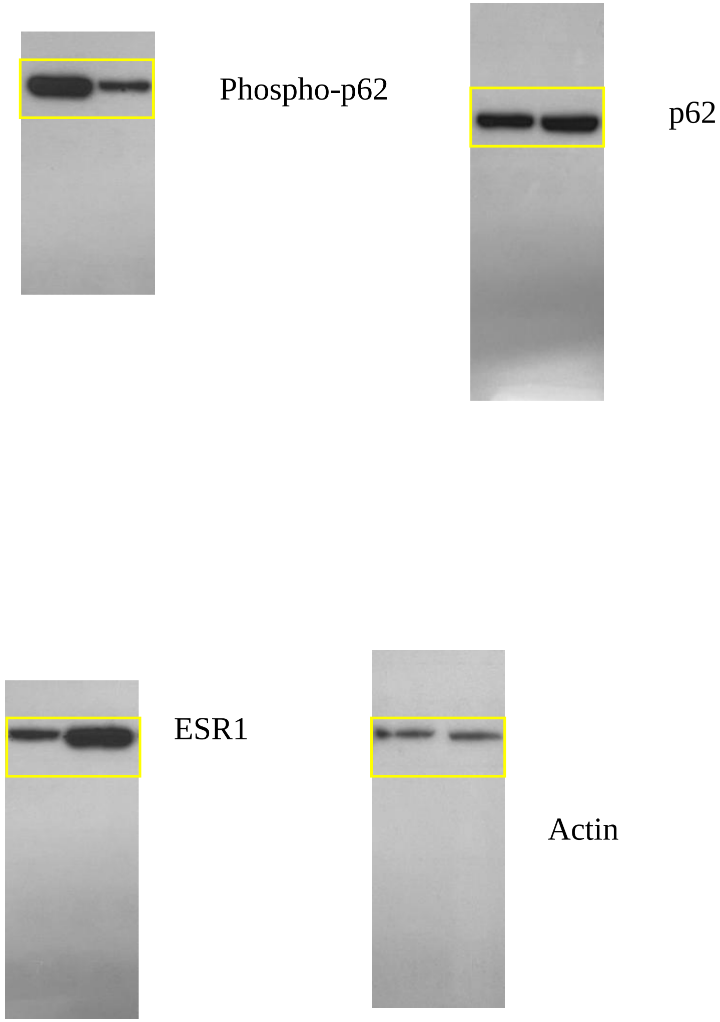

Fig. 6G

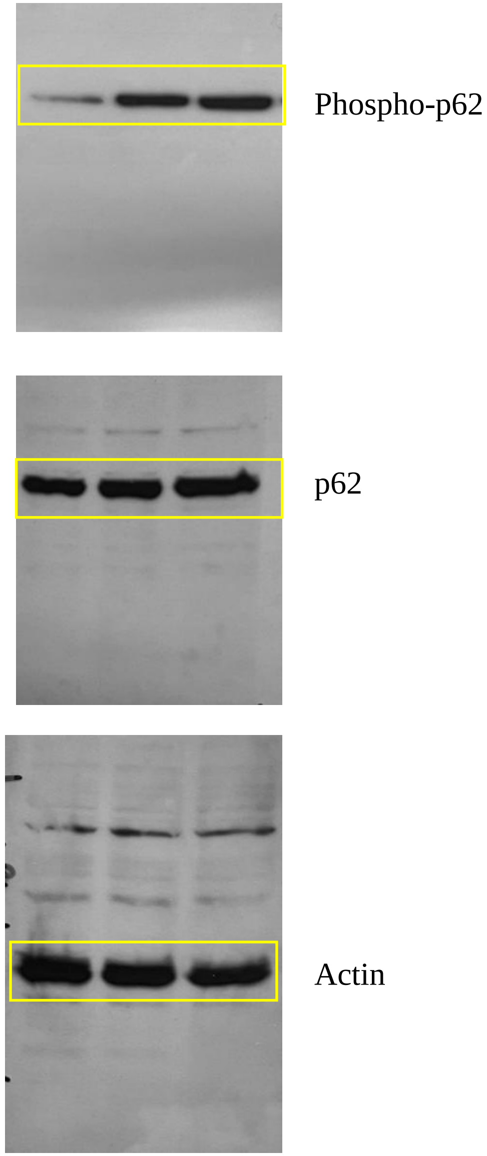

Fig. 6I

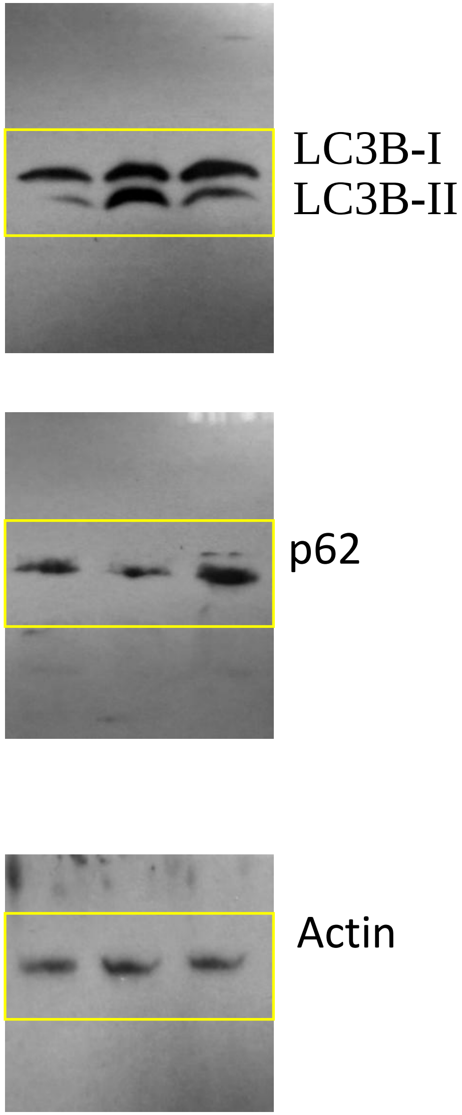

Fig. S2

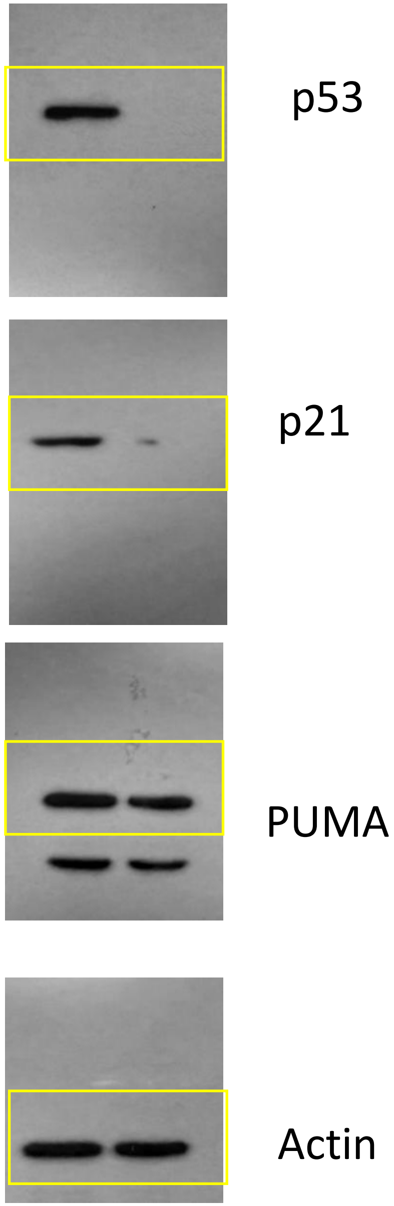

Fig. S3

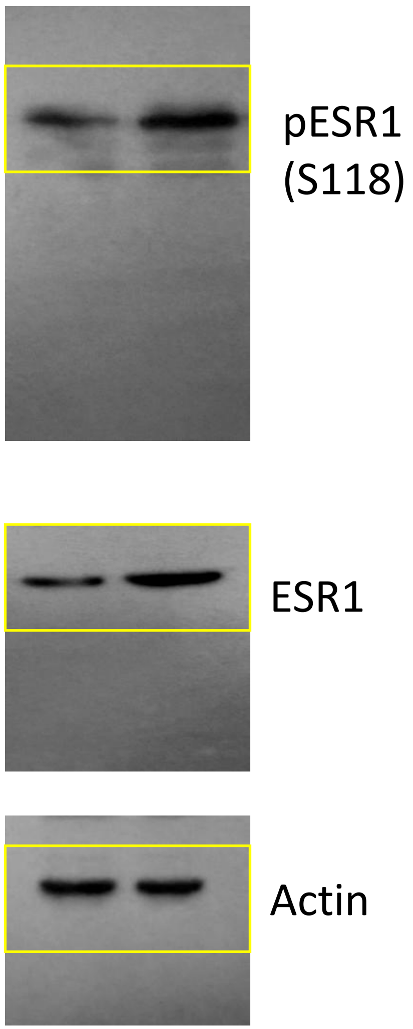

Fig. S4

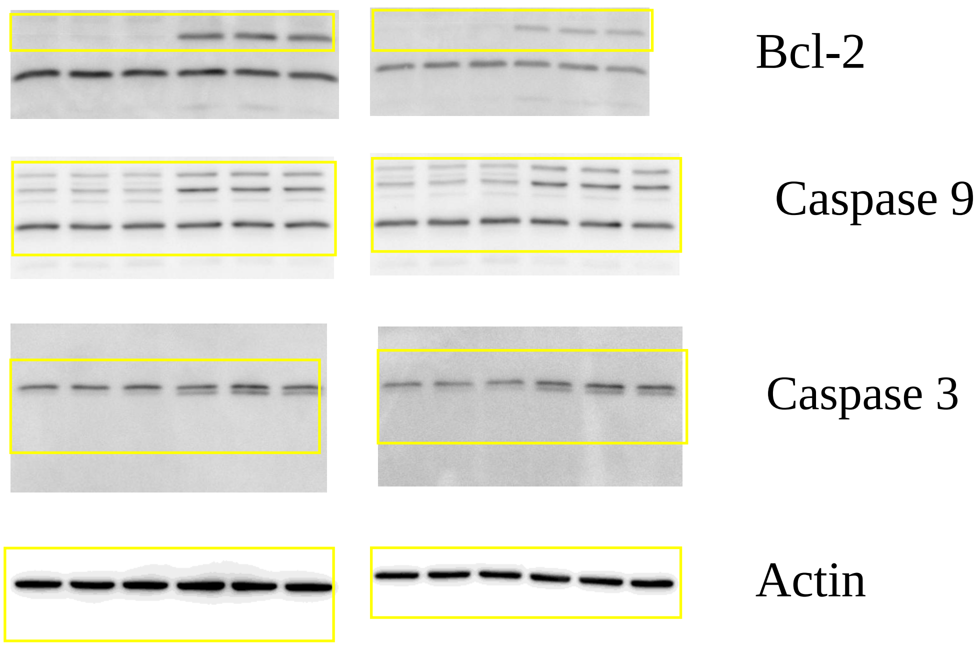

Fig. S5

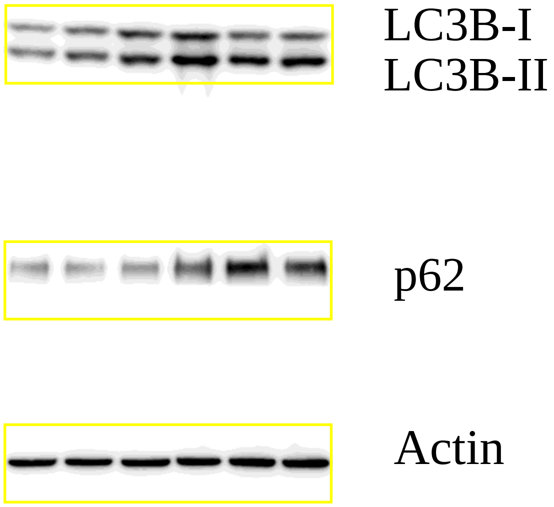

Supplement: Supplementary file 2 — Related Manuscript File [file 41420_2025_2755_MOESM2_ESM.pdf]
